# Environmental Impact of a Tooth Extraction: Life Cycle Analysis in a University Hospital Setting

**DOI:** 10.1111/cdoe.70003

**Published:** 2025-06-27

**Authors:** Paul Künzle, Ariadne Charis Frank, Sebastian Paris

**Affiliations:** ^1^ Department of Operative, Preventive and Pediatric Dentistry Charité – Universitätsmedizin Berlin Berlin Germany

**Keywords:** environmental footprint, green dentistry, life cycle analysis, life cycle assessment, sustainable dentistry, teledentistry, tooth extraction

## Abstract

**Objectives:**

The global impact of health care on the human environmental burden is enormous, but medical care is currently not realising the potential of sustainable practice. Similarly, dentistry and the various forms of dental treatment are not provided in a sustainable manner. This study focussed on quantifying the environmental burden of a standard dental treatment, specifically a tooth extraction, and on identifying the environmental impact of the process.

**Methods:**

A life cycle analysis was performed, simulating the entire process of a tooth extraction—including patient and staff travel, materials and washing/sterilisation procedures—using the software OpenLCA 1.11.0 and the database ecoinvent 3.9.1. The facilities, instruments and items used were those of Charité – Universitätsmedizin Berlin. For travel impact estimations, questionnaire data on travel modalities were gathered from patients and clinic staff. To evaluate possible approaches for more environmentally friendly processes, a change of the information/consent meeting from face‐to‐face to an online meeting was simulated.

**Results:**

The greatest single contributors to the environmental impact of an extraction procedure were travel, the production of steam (e.g., for sterilisation), electricity, soap, and waste. After normalisation, the process impact was highest on the categories: human toxicity (cancer effects and non‐cancer effects), freshwater ecotoxicity, resource use (energy carriers) and ionising radiation (human health). The total environmental impact was 13.8 kg CO_2_ equivalents, which compares to driving a distance of 56.3 km with a gasoline‐powered vehicle. The implementation of a digital consent process could reduce greenhouse gas emissions by 36.1% to 8.8 kg CO_2_ equivalents.

**Conclusions:**

Modelling the environmental impact of a dental extraction in a university hospital setting provided a detailed account of absolute and relative environmental impact contributions. The reduction of treatment‐related travel is the most effective measure to reduce the environmental impact of dental practice.

## 1 Introduction

Sustainability is one of the most pressing concerns of society, and climate change is one of the largest health threats that humanity currently faces [1, 2]. Climate change threatens global health by increasing both the incidence and severity of health conditions that are subject to climate and weather influences [3]. Potential negative climate change effects on health are: deaths due to higher temperatures, decreasing air quality [3], and greater exposure to pathogens threatening food safety and adequate nutrition [3]. Finally, mental health problems and workplace absenteeism may increase, caused by disruptions of the local environment, the associated stress and so‐called eco‐anxiety, a term used to describe stress caused by environmental turmoil [3, 4, 5].

To mitigate these outcomes, international agreements were made, such as the Sustainable Development Goals [6], the Paris Agreement [7], the European Green Deal [8], and the European Climate Law [9]. The European Union (EU) aims to reduce greenhouse gas (GHG) emissions from the 1990 level by 55% by 2030, and to become a climate‐neutral continent by 2050 [10]. At present, healthcare processes are not focused on environmental sustainability, but the healthcare system contributes significantly to society's GHG emissions. In the United States (US), the healthcare sector contributes 8.5% [11] to the national climate footprint, in Germany and the United Kingdom (UK), the shares amount to 5.2% [12] and 4% [13], respectively. Other sectors like transport or electricity and heat account for 29.1% and 32.0% (US), 20.2% and 34.3% (Germany) and 24.6% and 20.9% (UK) [14], which underscores the importance of the healthcare sector for GHG emissions.

As the impact of climate change becomes increasingly evident [15], careful attention must be given to changes needed to ensure sustainable oral health care. The World Dental Federation published a sustainability toolkit to help dental assistants and dentists implement a more sustainable practice of dentistry [16], and released a consensus statement on sustainability in dentistry summarising overarching sustainable dentistry practice guidelines [17, 18]. Dentistry contributes to climate change through inadequate disease prevention schemes, travel [19, 20], dental materials, the use of plastic packaging and single‐use plastics [18], dental products and procurement, and the use of energy [21]. While oral examinations account for the largest share of dental treatments, invasive and preventive treatment services follow, dependent on insurance benefits [22].

To analyse the causes of unsustainable practice, the environmental impact of all aspects of the treatment process must be examined in detail. Treatments vary significantly among countries, and procedures may entail different steps. In addition, an assessment of the provider environment—be it a private practice, an ambulatory healthcare centre, a university clinic or some other site of dental practice—is essential. Subsequently, savings potentials can be identified and optimisation measures considered.

The present study had two aims. The first aim was to analyse the environmental impact of a dental standard procedure, specifically a tooth extraction treatment, and to assess the relative contribution of its primary contributing components of the process. As travel of patients and personnel is a major contributor to the environmental impact of medical treatments, the second aim was to model how process modifications could reduce the need for travel.

## 2 Methods

In this study, a life cycle assessment (LCA) was employed to measure the environmental footprint of a tooth extraction. The LCA offered, after accounting for resource inputs and outputs and energy used, the opportunity to estimate the environmental impact of the product. Modelling could be performed either cradle‐to‐gate, that is from raw material to leaving the gates of the factory, or cradle‐to‐grave, from raw material to the end‐of‐life of the product (modelling the entire life cycle). For the modelling of procedures, parts of a treatment were regarded in a cradle‐to‐grave life cycle environmental impact analysis (e.g., for disposable and reusable products in a dental examination or a root canal treatment) [23, 24]. Additional processes, which by nature were not applicable for a cradle‐to‐grave analysis (such as healthcare travel), were modelled independently in the analysis.

The International Organization of Standardization has a formulated guideline for the process, ISO 14040:2006 [25], which dictates four stages. In stage one, the goal and scope definition, the system boundary, the product system and the functional unit were defined. Stage two comprised the collection of raw data, which were classified as inputs or outputs and quantified for the generation of a life cycle inventory (LCI). These data could then be translated to values that correspond to impact categories in the life cycle impact assessment (LCIA), stage three. A plethora of different impact categories exist and were evaluated during the assessment, such as resource use of energy carriers, minerals and metals, acidification, freshwater ecotoxicity and eutrophication, human toxicity (cancer effects) as well as ionising radiation and climate change. The findings were then analysed and interpreted in stage four, life cycle interpretation. In addition, values were normalised and weighted. For normalisation, impact category values were standardised using a reference value to compute their impact. Weighting was performed using an independently defined impact factor [26]. Categories that combined accounted for at least 80% of the entire impact were deemed to be of most significance [27].

### 2.1 Goal, Scope and System Boundaries

An attributional LCA was used employing the ISO 14040:2006 guidelines for the life cycle [25]. The study was designed to follow the scope ‘cradle‐to‐grave’ where applicable, including the production of dental instruments, any form of transport of patients, staff and materials, the use of involved materials, the waste management of disposables and preparation of reusable products (i.e., disinfection and laundry) and energy use (for extraction kit assumptions and inventory, see Appendix [Link], [Link], [Link] and 6). The travel modalities for staff and patients were obtained through a random survey of 97 patients and 26 staff members in the clinic. All surveyed participants confirmed their consent to participate in this study.

**FIGURE 1:**
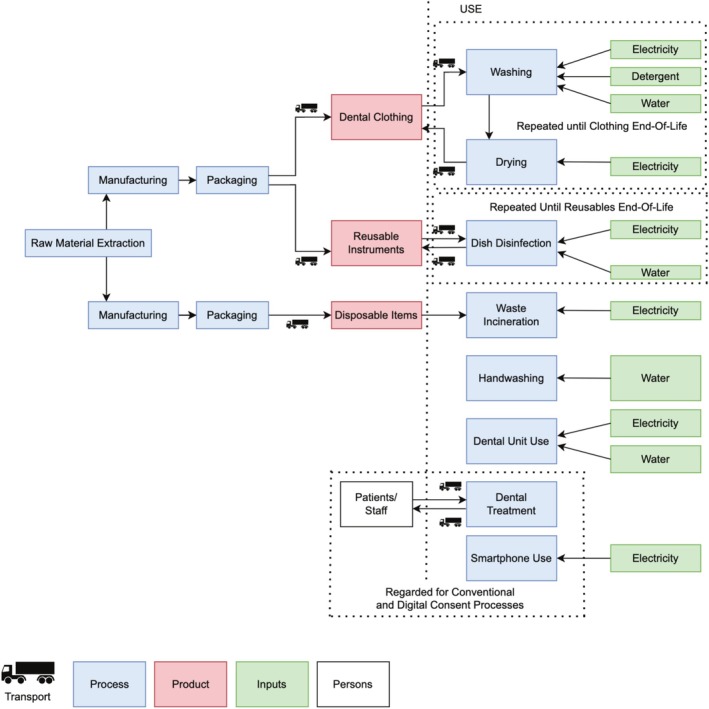
System boundary used in this modelling. For the first part of the assessment, a dental extraction with its conventional consent process was modelled. In the second part, a dental extraction with a digital consent process using smartphones was modelled.

The system boundary for modelling the environmental impact of the conventional procedure (Scenario A) included all materials and procedures relevant to the overall process. For scenario A, the process of a tooth extraction was modelled to include two appointments: the first to discuss the treatment with the patient and obtain their consent, and a second appointment for the actual treatment itself. In many countries, information and consent to the procedure are legally required at a separate appointment with sufficient time for reflection between the consultation and the actual procedure. All other appointments (e.g., for aftercare) were not included in this study. In a second scenario B, it was simulated that the first appointment was held digitally to avoid travel, which required the use of digital devices at the system boundary but reduced the amount of travel otherwise required to and from the treatment site. For system boundaries, see Figure 1. Any further appointments (e.g., necessary for follow‐up care) were not regarded in this study. Administration and accounting, any construction and heating of buildings, manufacturing and transport of greater machines and servicing (e.g., lights, dental units etc.) were excluded. For clarification of the scope of the study and presumptions made, see Appendix 1 and 2.

### 2.2 Life Cycle Inventory

The collection of all data for the presented life cycle analysis was performed for the treatment and its associated processes at Charité – Universitätsmedizin Berlin. This step was referred to as the creation of an inventory. The content of an extraction kit was established, and the average weight of each instrument was calculated for subsequent use. This was done by weighing ten items of each instrument [scale accuracy: 1.0 g (large scale)/0.3 mg (fine scale)]. Managing staff members of Charité – Universitätsmedizin Berlin helped with approximating the average lifetime of the various instruments, and consensus was found for the lifetime of most materials. Where interviewees disagreed, the shorter lifetime was assumed. Relative weights were used according to the approximated lifespan of one item. To analyse procedures such as examinations, waste disposal and dishwashing/laundry, it was necessary to define specific operating parameters. Water and energy consumption data for cleaning machines used, such as the dryer, washing machine and dishwasher, were retrieved from the manufacturer or responsible facility management personnel. Data to model the compressor and the dental unit consumption were drawn from Borglin et al. [23]. The transport distances were estimated based on the locations of the manufacturing sites and Charité – Universitätsmedizin Berlin. The distance between a local distributor in Berlin and the clinic was not considered. For transport calculation, a web‐based application, Searates, was used [28]. Single‐use materials were disposed of through incineration. Waste transportation was not included in the analysis. Life cycle inventory components and further information on used materials and machines are provided in Appendix 2.

Mean travel data for both staff and patients were derived from the surveys. A total of three data sets from patients and four sets from staff members were incomplete and therefore excluded from the analysis. For clarification, the life cycle inventory for included dental travel is shown in Appendix 3. Next, different scenarios with individual system boundaries were modelled for the conventional and the digital consent process requiring separate modifications (Scenarios A and B, see Appendix 4). Patient travel was fully included in the analysis, whereas staff travel was only partially regarded since staff perform multiple procedures per trip to and from work. All inventory data were then combined to form life cycle inventories for the conventional and for the digital consent process, and a supplemental analysis without dental travel. These are available in Appendix 5.

### 2.3 Life Cycle Impact Assessment (LCIA)

For the present study, the software OpenLCA, version 1.11.0 was used. For the LCA, the program ecoinvent, version 3.9.1, was used, including country‐specific data. The assessment was performed adhering to ISO 14040:2006. The data of this LCA were then modelled with the appropriate database processes. The analysis of the treatment included 16 environmental impact categories, as advised by the European Product Environmental Footprint (PEF) harmonisation initiative, available in Appendix 6. The PEF harmonisation initiative was developed to harmonise LCAs. The initiative comprises a selection of impact categories that can be used to evaluate the environmental burden of different processes [27]. After calculating the impact results, normalised data were gathered to determine the impact categories of greatest effect (using PEF adjusted to per capita global equivalents). Individual process contributions to impact categories of the conventional and digital consent process modellings and the supplementary analysis without dental travel are available in Appendix 7.

## 3 Results

### 3.1 First Aim: Analysis of the Standard Process

In the present modelling, one extraction procedure following the conventional consent process produced 13.8 kg CO_2_ equivalents, which translates to the use of an average gasoline‐powered vehicle for a distance of 56.3 km [29]. For all categories, the individual impact results were set in relation to the annual emission of one person. After normalisation of the calculated results, the impact categories a tooth extraction contributed most to were human toxicity (cancer effects), freshwater ecotoxicity, human toxicity (non‐cancer effects), ionising radiation and resource use (energy carriers). The main product categories and their contribution to the total burden of a dental extraction per impact category are shown in Appendix 7.

**FIGURE 2:**
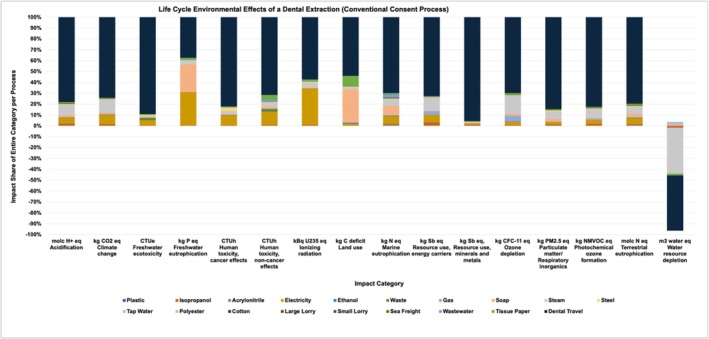
Environmental effects of a dental extraction across 16 impact categories are displayed [23, 30]. This analysis includes dental travel using a conventional consent process.

Dental travel was the strongest contributor to all categories (Figure 2). The production of steam for instrument sterilisation was the largest contributor to water resource depletion and a contributor to several other categories as well. The production of electricity also tremendously impacted most categories, except for land use, resource use (minerals and metals) and water resource depletion. The production of soap had a considerable effect on land use, freshwater eutrophication and marine eutrophication. The fabrication of tissue paper contributed to impact categories land use and human toxicity (non‐cancer effects) the most. The use of materials resulted in the production of waste, which affected multiple impact categories, most importantly freshwater ecotoxicity and human toxicity (non‐cancer effects). Tissue paper also had a sizable effect on many categories, including land use and human toxicity (non‐cancer effects).

Steel had a sizable effect on human toxicity (cancer effects). The use of the disinfection agent isopropanol had a minor impact on multiple categories, including resource use (energy carriers) and acidification; similarly, the use of gas had minor impacts on ozone depletion and resource use (energy carriers). Any form of instrument transport to the sterilisation facility (small lorry), laundry transport (large lorry) and sea freight had only minor impacts on the overall emission balance. Similarly, the production of textiles for pants, shirts and coats (dentist and assistant wear), the production of plastic, tap water, wastewater, gloves (acrylonitrile) and the disinfection agent ethanol had only minor impacts. The details of the life cycle impact assessment for one dental extraction using the conventional consent process (Scenario A) are depicted in Figure 2. For an analysis of the contributors to a dental extraction without dental travel, see Appendix 7.

### 3.2 Second Aim: Analysis of Process Change

**FIGURE 3:**
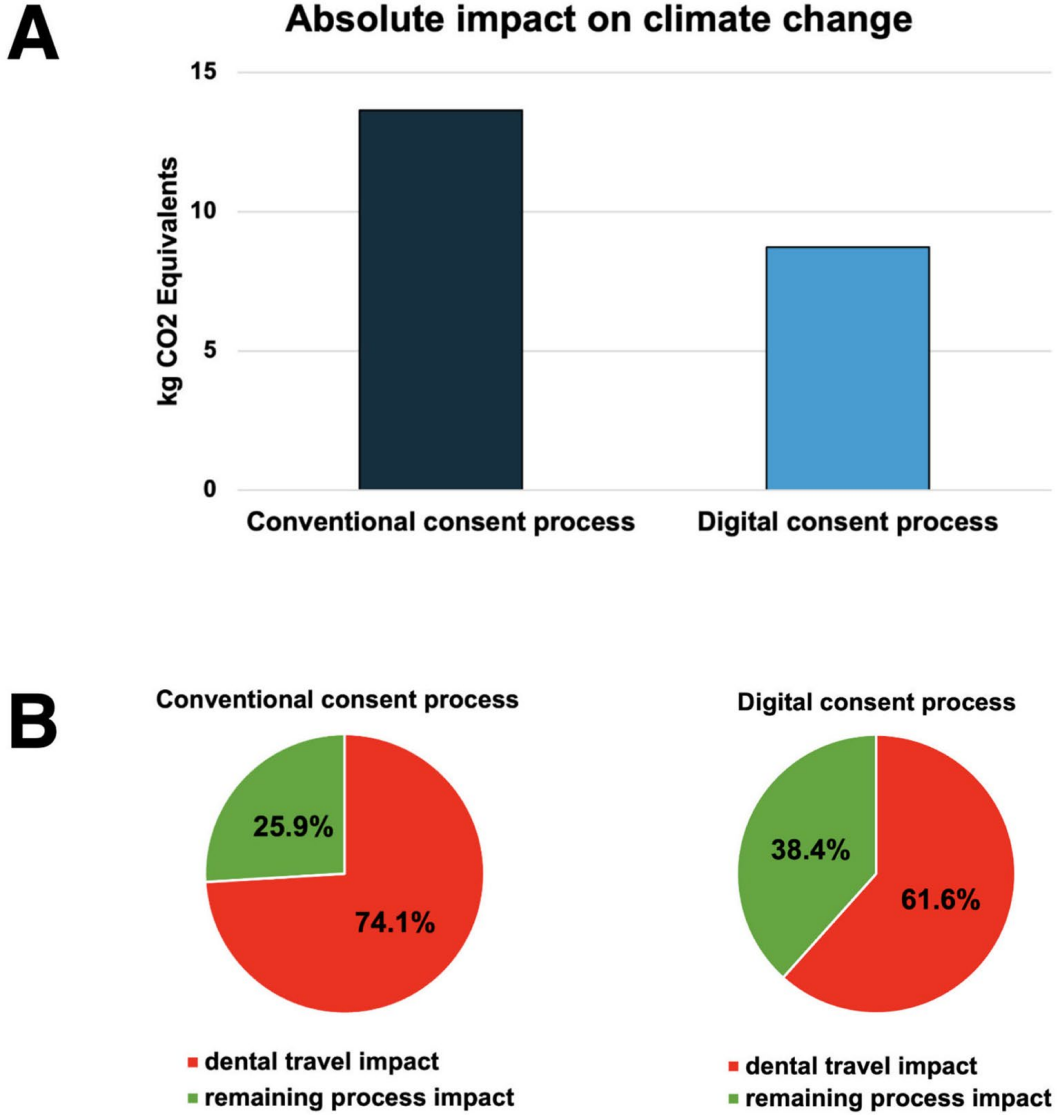
Climate change reduction potential of digital consent processes. Scenario A (conventional): Absolute results of environmental impact category 'climate change' using different consent processes. Scenario B (teledentistry): Relative share of dental travel in the entire process.

Compared to Scenario A, the use of a digital consent process to avoid a second on‐site appointment (Scenario B) significantly reduced carbon dioxide emissions. This way, 36.1% less CO_2_ equivalents were emitted, reducing the carbon footprint to a total of 8.8 kg CO_2_ equivalents (Figure 3A). Similarly, the relative share of dental travel of the entire carbon dioxide emission was reduced for both conventional and digital consent processes (Figure 3B). The differences in consent discussions are described in Appendix 4, life cycle inventories are shown in Appendix 6 and the full data of the life cycle impact assessment are displayed in Appendix 7.

**FIGURE 4:**
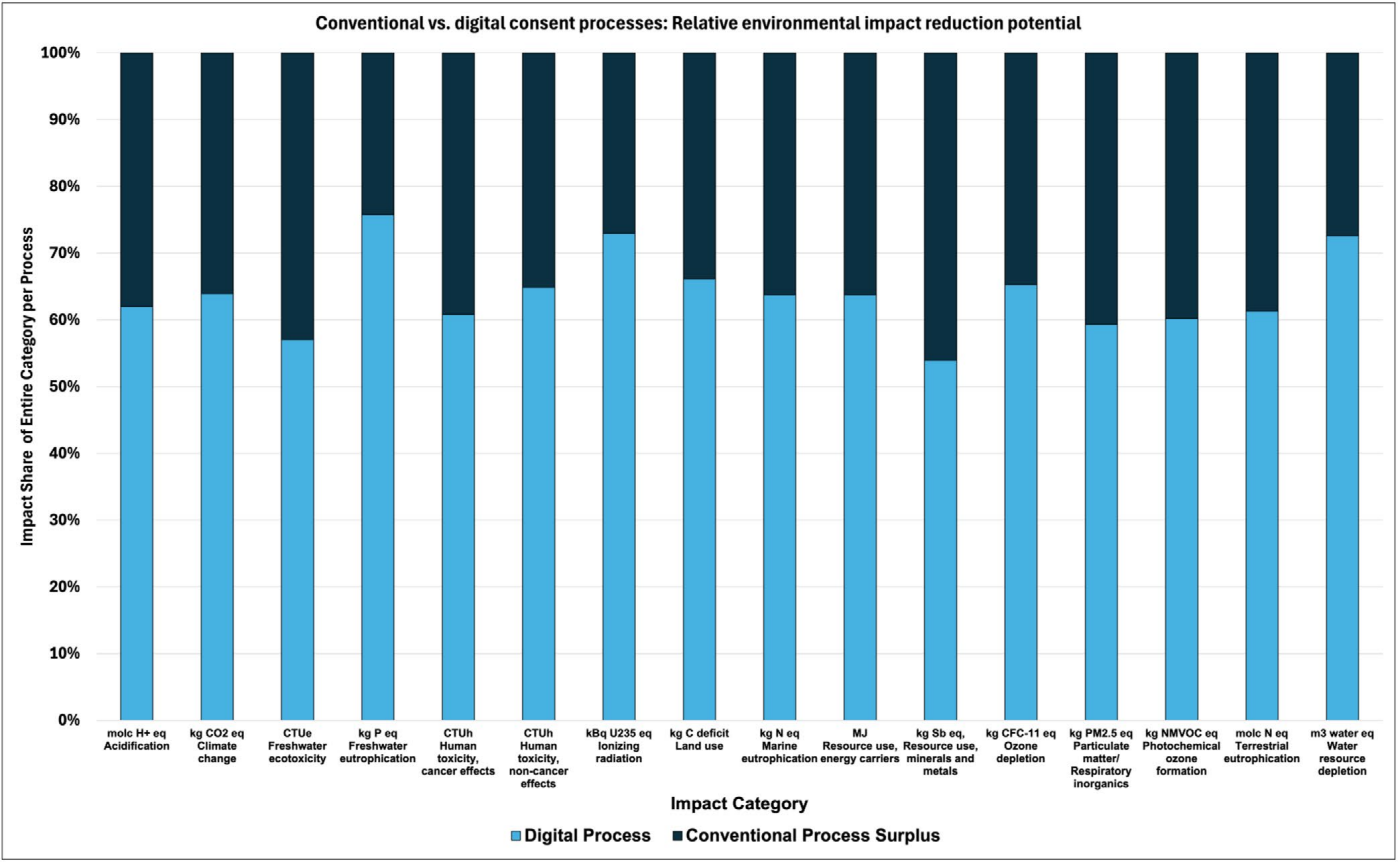
Decrease potential of the environmental footprint using a digital consent process. Impact categories were chosen as described before [23, 30]. In this analysis, the relative impact per category is shown.

In addition to the comparison of CO_2_ emissions and the relative impact on climate change, the overall reduction potential of a digital consent process was calculated for each individual impact category. The relative contribution to each impact field was plotted and a reduction of the environmental impact was found across all analysed categories. For most categories, the relative impact reduction reached over 30% per category, pointing to a diverse individual potential for environmental impact reduction (Figure 4).

## 4 Discussion

The aim of the study was to model the environmental impact of a tooth extraction in a dental clinic in a metropolitan area as one of the most common dental treatments to gain insights into the sustainability of the treatment processes and also to provide approaches for the implementation of environmentally optimised dental care. Dental travel, that is travel to and from the location of dental treatment, was found to make up a decent part of the environmental impact. Reducing the need for travel by carrying out the consent process in an online consultation could significantly reduce the environmental impact of the overall process in this simulation.

This study is the first that focused on the environmental impact of a tooth extraction, across several categories. Since extraction is one of the most frequently performed dental procedures worldwide, the procedure not only accounts for a substantial share of the environmental impact caused by dentistry [31], but optimising procedures could also contribute to a considerable reduction of the dental environmental footprint. The modelling accounted for the entire workflow of a dental extraction in a university clinic. While normalised findings for several impact categories are minimal, tooth extractions are a frequently performed dental procedure [32] and hence individual contributions can mount quickly. The underlying data were generated with attention to detail, including the conduction of surveys among staff and patients at the university clinic. Treatment associated with travel of patients and staff was found to make up a decent part of the environmental impact, which is in accordance with previous studies in different settings [31]. Although the process was modelled for a university clinic, the results are easily transferable to other dental facilities and smaller practices in a metropolitan environment, as their processes differ only minimally from those of a university clinic. However, due to the large impact of treatment‐related travel, the results would likely differ for rural areas or areas underserved by dentists.

Naturally, a modelling approach simplifies the situation of the real world. Hence, some parameters that may have an influence on a dental treatment process lie outside the system boundary of a life cycle analysis and were therefore not regarded. Moreover, the parameters used can only approximate the individual dental extraction, which can deviate in its use of materials and resources.

A limitation of this study is that it does not include parameters of a dental treatment that go beyond the treatment itself. For any treatment, the production, transport to the clinic, and utilisation of servicing and greater machines (e.g., lights, heating blocks) and their construction and maintenance are required, among many other influencing factors, and were not included in this study. The *in silico* data provide approximations of the environmental implications of a tooth extraction. However, since the study was conducted at a university clinic, in a large capital city and a wealthy member state of the European Union adhering to state‐of‐the‐art treatment procedures, the finding may not be immediately applicable towards other environments and have finite external validity. For example, the operational efficiency during instrument sterilisation and laundry of clothing [33] in one of the largest university hospitals in Europe is likely higher than in private practice, which often employs smaller scale processes. Also, in many countries, especially those with more limited resources, a separate appointment for consultation and consent is not legally required, so that a substantial proportion of the travel‐related influences are eliminated from the outset [34]. Also, in many settings, less instruments for a standard tooth extraction are employed by using separately sterilised instruments instead of instrument trays or an osteotomy box as in this study. The results should therefore be applied with caution if used to draw conclusions under other circumstances.

The tooth extraction was chosen here as a regularly performed treatment process that is relevant across private dental practices and universities. Previous studies assessed the environmental impact of dental procedures and materials [23, 24, 35, 36], an entire dental practice [37], and dental procurement [23]. The finding that, excluding dental travel, steam and electricity, soap is a strong contributor to the environmental burden of a dental extraction was identified in previous studies [23, 24, 35, 38]. Although the relevance of dental travel for the environmental impact of dental procedures was shown in previous studies [19, 20], the present study is the first to analyse the impact of a modification of the process to reduce travel‐associated environmental impacts. Telemedicine was shown in a different medical setting to reduce GHG emissions [39], and further data could also help assess the impact of regular dental prevention schemes [40, 41].

Survey‐based data on dental travel have not previously been incorporated into LCA studies. Dental travel time to different dental providers was analysed in the past [42] and the travel time was found to have increased for rural households over almost two decades, exacerbating healthcare provision shortages and further increasing travel‐related environmental impact contributions [43]. The promotion of remote clinical consultations was previously identified as a key strategic principle for the provision of environmentally sustainable oral health care [18]. While a digital consent process may be implemented in a medical and dental context, there are barriers to the immediate implementation [39]. Importantly, it needs to be ensured that the consent process withstands judicial objections and procedural complaints. Even conventional informed consent processes showed only mediocre understanding of participants in a clinical context [44]. Any virtual consultation needs to yield a data set which can be used for a decision‐making process that is as good as the conventional in‐person procedure. Hence, since teledentistry adds another level of complexity to the informed consent process through less physical proximity to the responsible dentist, first, the process itself needs to be improved before it can be transferred to a digital format. This study evaluated the reduction potential of teledentistry in comparison to an existing, conventional treatment process.

Tooth extractions are often the last resort in dental treatments and can increasingly be avoided through preventive and minimally invasive dentistry [45]. Therefore, preventive measures appear at first glance to offer the best approach for minimising the environmental impacts of dental procedures [17, 18, 46]. However, given the high proportion of travel in the dental environmental footprint, regular check‐ups and preventative visits, in particular—although certainly sensible from a preventive and medical perspective—can contribute significantly to the overall impact of the dental carbon footprint [31]. Therefore, health benefits of individual interventions may be associated with additional environmental costs. Future studies should therefore analyse not only the health benefits and the financial resources required to achieve them, but also the environmental costs to allow for a balanced assessment of the appropriateness of different interventions.

The present analysis yields insights into the sustainability of dental care provision. Overall, tooth extraction materials accounted for only a fraction of environmental impacts in this experimental set‐up. Although levers such as reducing dental travel provide a greater reduction of GHG emissions, more sustainable alternatives to conventional dental products are available. While infection control and proper decontamination procedures of instruments and professional clothing are mandatory [47], some of the established processes could be made more efficient. Reducing the number of contaminated instruments (which was 35 in this study) and providing these in smaller, separately sterilised containers, and ensuring each instrument's lifespan is met (and premature disposal hence prevented) could reduce the associated GHG emissions. As for travel, the economical restrictions in some countries may often result in a more sustainable process just due to the limitation of instruments. In the past, potential health benefits were considered so paramount that in Western economies hardly any economic costs were avoided to achieve an optimal result. With increasingly limited economic and ecological resources, it will be necessary to make a more differentiated assessment and to question the necessity of all partial aspects of complex treatment processes.

Moreover, sourcing instruments from countries that decarbonise their steel industry [48], replacing conventional soaps and detergents with sustainable alternatives such as biosurfactants [49], and extended use of sustainable fibre alternatives, for example, Tencel/lyocell or bacterial cellulose [50] and less use of unsustainable fibres such as cotton [23] may further decrease the detrimental effect on environmental impact categories in the future. Reusable dental bibs should not be used, since the need to decontaminate the bib after each use offsets any environmental advantages due to lengthy and energy‐intensive washing and sterilisation procedures [51]. Laundry heat energy reduction can only partially be compensated through prolonged washing times for achieving similar log reduction effects of certain microbials [52]. Similarly, at present, biosurfactants are not a potential alternative to conventional surfactants [53]. Operational efficiency, ensured by using washing and sterilisation machines at full load only, provides additional advantageous potential. Energy, if sourced from nuclear power plants, contributes substantially to the environmental impact category ionising radiation. Dental providers using non‐renewable energy sources could change energy providers to alleviate their environmental burden of dental treatments. At present, the literature does not provide sufficient evidence to claim that remote consultations are effective, safe and reliable measures in a clinical setting. Here, the necessary evidence and protocols would still need to be developed to implement a digital consultation service in the clinical routine.

Future research could focus on extending the LCA data available on dental treatment processes and investigate potential mechanisms for improvement. Further research could extend the system boundary of LCAs further and include (heavy) machinery and manufacturing of transport vehicles. Surveys among patients and staff could be undertaken on a regular basis to monitor how modes of transport change over time and, if implemented, actions for improved environmental sustainability of dental travel have the desired outcome [19]. For the teledentistry approach proposed in this study, the necessary legal framework and prerequisites would need to be established to enable the dental profession to perform remote clinical consultations in practice. Furthermore, the potential of prevention mechanisms to reduce dentistry‐associated GHG emissions should be analysed in detail to evaluate its potential as a cornerstone of sustainable dentistry. Overall, reducing the environmental impact of dental treatment procedures could assist in lifting the full potential of environmentally optimised dental care.

## 5 Conclusion

The environmental impact modelling of a dental extraction in a university hospital setting yielded a detailed account of absolute and relative environmental impact contributions. The reduction of treatment‐associated travel is a pragmatic measure to significantly reduce the environmental impact of the intervention.

## Author Contributions

P.K. and S.P. designed the study and were in charge of overall direction and planning. P.K. generated data for materials used, designed the model, the computational framework and analysed the data. A.C.F. designed a travel survey and generated data from patients and staff. P.K. wrote the original draft of the manuscript. P.K., A.C.F. and S.P. reviewed and edited the manuscript.

## Ethics Statement

Ethical approval for the staff and patient survey was obtained from the ethics committee of Charité – Universitätsmedizin Berlin (EA2/191/23).

## Conflicts of Interest

The authors declare no conflicts of interest.

## Supporting information


**Appendix S1** Supporting Information

**Appendix S2** Supporting Information

**Appendix S3** Supporting Information

**Appendix S4** Supporting Information

**Appendix S5** Supporting Information

**Appendix S6** Supporting Information

**Appendix S7** Supporting Information

## Data Availability

The data that supports the findings of this study are available in the supplementary material of this article.
