# Supplementary material for: Environmental Impact of a Tooth Extraction: Life Cycle Analysis in a University Hospital Setting
Source: Community Dent Oral Epidemiol. 2025 Jun 27;54(1):30–9. doi: 10.1111/cdoe.70003 (PMC12808852; doi:10.1111/cdoe.70003)
Supplement: Supplementary file 1 — Appendix S1 Supporting Information [file CDOE-54-30-s006.docx]

# Appendix 1. Extraction kit assumptions.

| List of assumptions |
| --- |
| A dental extraction was calculated to last 30 minutes |
| The dentist performs the examination with a dental assistant |
| The dental extraction modelled was assumed to be performed at Charité – Universitätsmedizin Berlin |
| Products external to Charité – Universitätsmedizin Berlin were transported in a large lorry, internally/within in a small lorry |
| Products produced outside Europe were first transported by a large lorry to the closest port, then by cargo ship to Hamburg port and finally by a large lorry to Berlin |
| All land transport was based on European transport |
| The packaging of dental equipment was excluded, no cardboard packaging was regarded in this analysis |
| The dentist and dental assistant use one set of clothing per day, consisting of a shirt, trousers and a coat. Shirt and trousers were made of 65 % polyester and 35 % cotton, coats were made of 67 % polyester and 33 % cotton, according to the manufacturer's description. Lifetime of the clothing: Trousers were expected to be used for 6 years, shirts for 3 years and coats for 10 years with 260 working days per year. It was assumed that both dentist and assistant wear a new set of clothing each day. |
| The dental unit was wiped down once with a paper towel and disinfectant after each extraction. 100 mL of surface disinfection (45 %, 2-propanol) and four paper towels were used per clean |
| Before meeting the patient, the dentist and the dental assistant washed and disinfected their hands. During the handwashing procedure, one liter of water, 10 g of hand soap and 5 mL of hand disinfection (75 % ethanol) were used. The dentist and assistant then proceed to wear gloves, size medium, for each procedure. Disposable products were discarded after a single use. All reusable stainless-steel products were disinfected in the dishwasher after use and subject to sterilization. |
| 12 examination kits were loaded in the dishwasher during each standard decontamination cycle. |
| Clothing was washed and dried in a large-scale facility in Sachsen-Anhalt, Germany. |
| The origin of products and transport thereof to Charité – Universitätsmedizin Berlin was only included for the core process of a dental extraction (i.e. materials used) and not for the provision of machinery for the washing/sterilization processes |
| All products entered the general waste stream at the end of their lifetime and were not classified as hazardous waste |
| General expenditures for buildings, e.g. heating, infrastructure construction and maintenance were not regarded. |
| Administration (e.g. scheduling) and accounting were not included in this study. |

**Table 1.** List of assumptions for the life cycle analysis of a dental extraction.

Assumptions were made for the definition of the system boundary.
