# Supplementary material for: Environmental Impact of a Tooth Extraction: Life Cycle Analysis in a University Hospital Setting
Source: Community Dent Oral Epidemiol. 2025 Jun 27;54(1):30–9. doi: 10.1111/cdoe.70003 (PMC12808852; doi:10.1111/cdoe.70003)
Supplement: Supplementary file 2 — Appendix S2 Supporting Information [file CDOE-54-30-s007.docx]

# Appendix 2. Extraction treatment assumptions.

Life cycle inventory

The full life cycle from production to utilization, including preparation processes such as laundry, washing and sterilization and waste management were included in this analysis for dental instruments (reusable and non-reusable) as well as staff laundry. The modeling also regarded the use of an equipped dental unit that also provides disposable materials. Power, water, gas and wastewater use/emissions were modelled for each machine used. Of note, due to water vaporization, the estimate for wastewater is lower than the total amount of water used. Out of the scope of this analysis was the construction of buildings, machines and electrical devices used. Dental travel was included for the modeling of two alternative scenarios, presented in Appendix 7.

Transportation

For the calculation of distances, the locations for each modelled provider of materials/services were used and connected with the location of the dental clinic of Charité – Universitätsmedizin Berlin. For the production of extraction kit materials, the location provided for a pair of steel tweezers was used for the modeling of the entire sourcing process. For the calculation of transport distances, the online tool Searates was employed ^1^.

Utilization

All single use products were subject to disposal after their use. Dental clothing was prepared in an external location, which provided data for laundry preparation in their facilities. Stainless-steel products were washed at the dental clinic in Steelco DS800 machines and sterilized in a different site of the Charité, Campus Benjamin Franklin, in Selectomat PL 9612 machines after use. Washing cycles modelled the preparation of 6 boxes per cycle, sterilization was modelled using 12 kits per cycle.

Procedural times

For a dental extraction, 30 minutes were approximated for the process. Data for the modeling were adapted from Borglin et al. and modified to fit the specification of the extraction process ^2^. The use of individual equipment is listed in the table below.

**Extraction kit**

| **Item** | **Quantity** | **Assumed lifespan** | **Material** | **Total weight (g)** | **Origin (proposed)** |
| --- | --- | --- | --- | --- | --- |
| Plastic Seal | 2 | 1 | Plastic | 0.81 | Sialkot, Pakistan |
| Steel box | 1 | 13000 | Stainless Steel | 1.12 | Sialkot, Pakistan |
| Steel lid | 1 | 13000 | Stainless Steel | 1.09 | Sialkot, Pakistan |
| Steel strainer | 1 | 13000 | Stainless Steel | 1.78 | Sialkot, Pakistan |
| Paper liner | 1 | 1 | Paper | 52.83 | Sialkot, Pakistan |
| Kidney basin | 1 | 13000 | Stainless Steel | 0.45 | Sialkot, Pakistan |
| Small cup | 1 | 13000 | Stainless Steel | 0.14 | Sialkot, Pakistan |
| Tweezers (anatomical) | 1 | 5200 | Stainless Steel | 0.06 | Sialkot, Pakistan |
| Tweezers (dental) | 1 | 1820 | Stainless Steel | 0.04 | Sialkot, Pakistan |
| Surgical tweezers | 1 | 1820 | Stainless Steel | 0.06 | Sialkot, Pakistan |
| Dental probe | 1 | 1300 | Stainless Steel | 0.03 | Sialkot, Pakistan |
| Dental mirror support | 1 | 13000 | Stainless Steel | 0.03 | Sialkot, Pakistan |
| Dental mirror | 1 | 520 | Stainless Steel | 0.09 | Sialkot, Pakistan |
| Dental curette | 1 | 260 | Stainless Steel | 0.46 | Sialkot, Pakistan |
| Heidemann scoop | 1 | 2600 | Stainless Steel | 0.04 | Sialkot, Pakistan |
| Beck hook (angled) | 1 | 13000 | Stainless Steel | 0.16 | Sialkot, Pakistan |
| Beck hook (pointed) | 1 | 13000 | Stainless Steel | 0.16 | Sialkot, Pakistan |
| Cheek displacer | 1 | 13000 | Stainless Steel | 0.15 | Sialkot, Pakistan |
| Mosquito clamp (u-shaped) | 1 | 5200 | Stainless Steel | 0.04 | Sialkot, Pakistan |
| Mosquito clamp (straight) | 1 | 5200 | Stainless Steel | 0.03 | Sialkot, Pakistan |
| Mosquito clamp (half-rounded) | 1 | 5200 | Stainless Steel | 0.04 | Sialkot, Pakistan |
| Needle holder | 1 | 5200 | Stainless Steel | 0.10 | Sialkot, Pakistan |
| Scalpel holder | 1 | 13000 | Stainless Steel | 0.04 | Sialkot, Pakistan |
| Scissors | 1 | 1820 | Stainless Steel | 0.04 | Sialkot, Pakistan |
| Raspatory | 1 | 10400 | Stainless Steel | 0.05 | Sialkot, Pakistan |
| Maxillary sinus probe | 1 | 1820 | Stainless Steel | 0.004 | Sialkot, Pakistan |
| Prichard curette | 1 | 13000 | Stainless Steel | 0.06 | Sialkot, Pakistan |
| Tamponade placer | 1 | 10400 | Stainless Steel | 0.04 | Sialkot, Pakistan |
| Elevator | 1 | 13000 | Stainless Steel | 0.05 | Sialkot, Pakistan |
| Sharp spoon (Hemingway) | 3 | 10400 | Stainless Steel | 0.45 | Sialkot, Pakistan |
| Diamond bur box incl. burs | 1 | 520 | Stainless Steel | 1.21 | Sialkot, Pakistan |
| Instrument holder | 1 | 520 | Plastic | 0.20 | Sialkot, Pakistan |
| Bein lever (short) | 1 | 13000 | Stainless Steel | 0.09 | Sialkot, Pakistan |
| Bein lever (long) | 1 | 13000 | Stainless Steel | 0.10 | Sialkot, Pakistan |

**Table 2.** Extraction kit assumptions for quantity, assumed lifespan, material, weight and origin of items used.

Instrument origins were assumed to be located in Sialkot, Pakistan as one of the world's largest hub for medical steel instrument production and confirmed origin from one manufacturer^3^.

**Dental clothing for personnel**

| **Item** | **Quantity** | **Assumed**  **lifespan (d)** | **Material** | **Origin (proposed)** |
| --- | --- | --- | --- | --- |
| Laundry pants (dentist) | 1 | 1560 | 35% Cotton, 65% Polyester | Shengze, China |
| Laundry shirt  (dentist) | 1 | 780 | 35% Cotton, 65% Polyester | Shengze, China |
| Laundry coat  (dentist) | 1 | 2600 | 33% Cotton, 67% Polyester | Shengze, China |
| Laundry pants  (dental assistant) | 1 | 1560 | 35% Cotton, 65% Polyester | Shengze, China |
| Laundry shirt  (dental assistant) | 1 | 780 | 35% Cotton, 65% Polyester | Shengze, China |
| Laundry coat (dental assistant) | 1 | 2600 | 33% Cotton, 67% Polyester | Shengze, China |

**Table 3.** Laundry process assumptions for dental personnel.

Laundry assumptions for dental personnel required in a dental extraction for quantity, material, weight and origin. Items listed are estimated for two dental professionals (1 dentist, 1 dental assistant) who work 8 h a day and perform 8 extractions per day. The lifespan was estimated by experienced dental personnel of Charité – Universitätsmedizin Berlin. The weight was divided by 8 prior to entry into OpenLCA for the lifespan adjustment.

**Laundry process**

| Material | Amount (g, conventional) | Amount (g, digital) |
| --- | --- | --- |
| Detergent | 3.71 | 3.09 |
| Power | 0.02 | 0.02 |
| Gas | 0.14 | 0.12 |
| Largest truck possible | 0.01 | 0.01 |
| Wastewater | 0.95 | 0.79 |
| Water | 1.05 | 0.88 |
| Cotton | 0.06 | 0.05 |
| Polyester | 0.12 | 0.1 |

**Table 4.** Laundry process assumptions.

Data for laundry processing were obtained from the laundry company Charité – Universitätsmedizin Berlin works with.

**Sterilization process**

| Material | Total amount |
| --- | --- |
| Transport Small Truck | 54.12 kg*km |
| Autoclave Sel. PL 9612, Steam | 5.4 l |
| Autoclave Sel. PL 9612: Power | 0.33 kWh |
| Autoclave Steelco DS800, Sel. PL 9612: Water | 13.75 l |
| Autoclave Steelco DS800, Sel. PL 9612: Power | 0.88 kWh |
| Steam | 5.4 l |
| Power (Total) | 1.22 kWh |

**Table 5.** Sterilization process assumptions.

Sterilization data were modelled using the processes established at Charité – Universitätsmedizin Berlin.

**Usage of material for dental extraction (dental chair)**

| Usage for Dental Unit, preparation etc. | LCI Database | Amount | Amount (adjusted to concentration) |
| --- | --- | --- | --- |
| Surface Disinfection, 45 % | 2-Propanol | 100 ml | 45 g |
| Paper Towels | Paper | 4 g | 4 g |
| Water | Tap water | 1 l | 2*/1** l |
| Soap | Hand soap | 20*/10** g | 20*/10** g |
| Hand Desinfection, 75 % | Ethanol | 10 ml | 0.0075*/0.00375** g |
| Examination gloves, 4 pc. = 6 g | Plastic | 12 g | 12*/6** g |
| Dental unit, water consumption per extraction | Water | 0.5 l | 0.5 l |
| Dental unit, power consumption per extraction | Power | 0.644 kWh | 0.644 kWh |

**Table 6.** Dental chair process assumptions.

Assumptions for the dental chair were modelled to be similar to a dental examination and therefore adapted from Borglin et al.^2^. The amount of surface disinfection and ethanol were in the modeling adjusted to their concentration.

*conventional consent process, **digital consent process

**Dental unit use**

| Use of a dental unit for a dental extraction | | |
| --- | --- | --- |
| Duration (min) | 30 | |
| Water usage (L) | 0.5 | |
| Machine (Power; W) | Procedural time (min) | kWh per extraction |
| Dental unit motor (400) | 2 | 0.013 |
| Dental light (40) | 30 | 0.018 |
| Unit screen (30) | 30 | 0.013 |
| Instrument light (2.5) | 0 | 0 |
| Suction (9000) | 2 | 0.3 |
| Compressor-operated machines (9000) | 2 | 0.3 |
|  | Total power consumed | 0.644 |

**Table 7.** Dental unit process assumptions.

Collection of procedural times, water and energy use and the calculated consumption for one dental extraction.

**Autoclave and washing machine**

| Machine | Company | Power (kW) | Use time (min) | Steam*/Water** consumption (l) | Energy consumption (kWh) |
| --- | --- | --- | --- | --- | --- |
| Autoclave | Selectomat PL 9612 | n/a | 66 | 30* | 2 |
| Washing machine | Steelco DS800 | 13.5 | 73 | 165** | 10.6 |

**Table 8.** Autoclave and washing machine process assumptions.

Energy and water consumption in the instrument sterilization process

**Laundry treatment process**

| Machine | Company | Power (kW) |
| --- | --- | --- |
| Washing Street | Kannegiesser PTV 85-16 SBR | 31 |
| Drying | Kannegiesser PS Plus 85 Turbo INT | 58 |
| Tunnelfisher | Kannegiesser XMT-3-Gas-Turbo D-R/M-Y | 30 |
| Folding machine | Kannegiesser FAX-01-A900 | 2.8 |

**Table 9.** Laundry treatment process assumptions.

Machines used for laundry processing of clothing used for a dental extraction

**Disposal/end of life**

All waste accumulated during and produced by the dental extraction was modelled to be incinerated. The transport process to the incineration facility was not modelled in this study.
