# Supplementary material for: Environmental Impact of a Tooth Extraction: Life Cycle Analysis in a University Hospital Setting
Source: Community Dent Oral Epidemiol. 2025 Jun 27;54(1):30–9. doi: 10.1111/cdoe.70003 (PMC12808852; doi:10.1111/cdoe.70003)
Supplement: Supplementary file 3 — Appendix S3 Supporting Information [file CDOE-54-30-s003.docx]

# Appendix 3. Life cycle inventory creation for patient and staff travel.

**Patient and staff travel assumptions**

| Patient and staff travel assumptions |
| --- |
| The system boundary entails the transport of one single dental patient and staff member from their home to the dental clinic. |
| Data from 20 queried staff members was included in this study, with the exclusion of 3 entries due to incomplete questionnaire entries. The cumulative amount for all included staff members is regarded in the inventory. |
| Data from 20 queried patients was included in this study, with the exclusion of 3 entries due to incomplete questionnaire entries. The cumulative amount for all included staff members was regarded in the inventory. |

**Table 10.** Patient and staff travel assumptions.

Life cycle inventories for dental travel were created from questionnaire results (see below) and subsequently integrated with the dental extraction inventory to yield a life cycle inventory for the conventional and digital consent process (see Appendix 5).

**Staff travel life cycle inventory**

| Mode of transportation | Amount (total) | Amount (p.p.) | Unit | LCI Database Process | LCI Database |
| --- | --- | --- | --- | --- | --- |
| Pedestrian | \| 3.85 \| \| --- \| | 0.18 | km | *no database process selected* | ecoinvent 3.9.1 |
| Motor scooter | 0.41 | 0.02 | p*km | transport, passenger, motor scooter |  |
| Electric scooter | 4.0 | 0.18 | km | transport, passenger, electric scooter |  |
| Bicycle, electric | 6.12 | 0.28 | p*km | transport, passenger, electric bicycle |  |
| Bicycle | 16.7 | 0.73 | p*km | transport, passenger, bicycle |  |
| Small car, petrol, EURO 2 | 0.6 | 0.03 | km | transport, passenger car, small size, petrol, EURO 3 |  |
| Small car, petrol, EURO 4 | 40.9 | 1.86 | km | transport, passenger car, small size, petrol, EURO 4 |  |
| Small car, petrol, EURO 6 | 0.68 | 0.03 | km | transport, passenger car, small size, petrol, EURO 5 |  |
| Medium-sized car, petrol, EURO 4 | 6.61 | 0.30 | km | transport, passenger car, large size, petrol, EURO 4 |  |
| Medium-sized car, diesel, EURO 4 | 12.0 | 0.55 | km | transport, passenger car, large size, diesel, EURO 4 |  |
| Large-sized car, petrol, EURO 1 | 3.0 | 0.14 | km | transport, passenger car, large size, petrol, EURO 3 |  |
| Large-sized car, petrol, EURO 6 | 9.2 | 0.42 | km | transport, passenger car, large size, petrol, EURO 5 |  |
| Omnibus | 0.56 | 0.03 | p*km | transport, passenger coach |  |
| Tram | 10.94 | 0.5 | p*km | transport, tram |  |
| Regional and urban (underground) trains | 170.41 | 7.75 | p*km | transport, passenger train |  |

**Table 11.** Staff travel life cycle inventory

After correspondence with the ecoinvent database support, there is no specific process available for German urban (underground) railway services "U-Bahn" and "S-Bahn". Adhering to their suggestion, railway transport was modelled with the same process used for regional trains. For pedestrians, no process was used in this modeling.

**Patient travel life cycle inventory**

| Mode of transportation | Amount | Amount p.p. | Unit | LCI Database Process | LCI Database |
| --- | --- | --- | --- | --- | --- |
| Pedestrian | \| 44.3075 \| \| --- \| | 0.47135638 | km | *no database process selected* | ecoinvent 3.9.1 |
| Motorcycle | 57 | 0.60638298 | p*km | transport, passenger, motor scooter |  |
| Electric scooter | 4.15 | 0.04414894 | km | transport, passenger, electric scooter |  |
| Bicycle, electric | 3 | 0.03191489 | p*km | transport, passenger, electric bicycle |  |
| Bicycle | 66.92 | 0.71191489 | p*km | transport, passenger, bicycle |  |
| Small car, petrol, EURO 4 | 73.3 | 0.77978723 | km | transport, passenger car, small size, petrol, EURO 3 |  |
| Medium-sized car, petrol, EURO 4 | 186.9 | 1.98829787 | km | transport, passenger car, small size, petrol, EURO 4 |  |
| Medium-sized car, petrol, EURO 6 | 14 | 0.14893617 | km | transport, passenger car, small size, petrol, EURO 5 |  |
| Medium-sized car, diesel, EURO 4 | 7.7 | 0.08191489 | km | transport, passenger car, large size, petrol, EURO 4 |  |
| Medium-sized car, diesel, EURO 6 | 11 | 0.11702128 | km | transport, passenger car, large size, diesel, EURO 4 |  |
| Medium-sized car, gas | 153 | 1.62765957 | km | transport, passenger car, medium size, natural gas, EURO 4 |  |
| Medium-sized car, electric | 10.5 | 0.11170213 | km | transport, passenger car, electric |  |
| Large-sized car, petrol, EURO 4 | 8 | 0.08510638 | km | transport, passenger car, large size, petrol, EURO 4 |  |
| Large-sized car, diesel, EURO 4 | 7 | 0.07446809 | km | transport, passenger car, large size, diesel, EURO 4 |  |
| Omnibus | 34.86 | 0.37085106 | p*km | transport, passenger coach |  |
| Tram | 21.075 | 0.22420213 | p*km | transport, tram |  |
| Regional and urban (underground) trains | 518.4875 | 5.51582447 | p*km | transport, passenger train |  |

**Table 12.** Patient travel life cycle inventory.

For the modeling of patients using hybrid cars, the distance was split evenly and added to the processes for medium-sized electric and petrol-fueled cars.
