# Supplementary material for: Environmental Impact of a Tooth Extraction: Life Cycle Analysis in a University Hospital Setting
Source: Community Dent Oral Epidemiol. 2025 Jun 27;54(1):30–9. doi: 10.1111/cdoe.70003 (PMC12808852; doi:10.1111/cdoe.70003)
Supplement: Supplementary file 4 — Appendix S4 Supporting Information [file CDOE-54-30-s002.docx]

# Appendix 4 Consent process assumptions and inventory.

## Scenario modeling: assumptions and system boundary

The system boundary here includes the full extraction process as well as travels to and from the clinic for patients and staff involved in the dental extraction consent process and treatment. The system boundary excludes all requirements for the production of a smart phone and only regards the power use required for its use.

## Life cycle inventories for Scenarios A and B

For the simulation of medical informed consent processes, two alternative scenarios were modelled. Travel distances were generated through questionnaire-based assessments and results for their impact are reported in Appendix 6.

In Scenario A, a patient physically comes to Charité – Universitätsmedizin Berlin and participates in a dentist-patient conversation that aims to enable the patient to decide for or against treatment procedures. Here, four full travel journeys are needed for the patient (journey to and from the clinic for two appointments) and eight partial travel journeys are needed for the staff (journey to and from the clinic, modified by a factor to account for 15 and 30 min of work time allocated for the consent process and extraction, respectively).

In Scenario B, the dentist-patient conversation is held virtually, modelled by both the patient and the dentist using smart devices to communicate with each other. Here, only two full travel journeys are needed for the patient (journey to and from the clinic for one appointment), while six partial travel journeys are needed for the staff. In addition, the power used for the consent process is regarded in this scenario. For the consent appointment, the dentist is modelled to partake in the consent discussion without the dental assistant. In the treatment appointment, the patient, dental assistant and dentist meet at the clinic as before.

**Scenario A**: Conventional informed consent process

| **Item** | **Quantity** | **Division Factor** | **Quantity (adjusted)** |
| --- | --- | --- | --- |
| Travel (Staff) | 8 | 0.09375 | 0.75 |
| Travel (Patient) | 4 | 1 | 4 |

**Table 13.** Staff and patient travel assumptions for a conventional informed consent process.

Assumption: Patients have to physically to travel to Charité – Universitätsmedizin Berlin to perform medical consent appointments. Modes of transportation used according to Appendix x and x. The division factor accounted for only partial use of the daily time of work of dental professionals allocated for the consent process (15 min for the consent process and 30 min for the extraction procedure).

**Scenario B**: Sustainably optimized informed consent process

| **Item** | **Quantity** | **Capacity (Wh, h)** | **Use Time** | **Power required** |
| --- | --- | --- | --- | --- |
| Phone | 2 | 12, 8 | 15 min each | 0.75 Wh |

**Table 14.** Phone assumptions for a digital informed consent process.

| **Item** | **Quantity** | **Division Factor** | **Quantity (adjusted)** |
| --- | --- | --- | --- |
| Travel (Staff) | 6 | 0.09375 | 0.5625 |
| Travel (Patient) | 2 | 1 | 2 |

**Table 15.** Staff and patient travel assumptions for a digital informed consent process.

Assumption: Dental personnel does not have to travel to Charité – Universitätsmedizin Berlin to perform medical consent appointments. The production of the phone is not included in this analysis. Phone capacity was modelled using publicly availably phone capacity information^1^. Again, the division factor accounted for only partial use of the daily time of work of dental professionals allocated for the consent process (15 min for the consent process and 30 min for the extraction procedure).
