# Supplementary material for: Environmental Impact of a Tooth Extraction: Life Cycle Analysis in a University Hospital Setting
Source: Community Dent Oral Epidemiol. 2025 Jun 27;54(1):30–9. doi: 10.1111/cdoe.70003 (PMC12808852; doi:10.1111/cdoe.70003)
Supplement: Supplementary file 5 — Appendix S5 Supporting Information [file CDOE-54-30-s004.docx]

# Appendix 5 Life cycle inventories for different scenarios (conventional and digital consent process, supplementary analysis).

**Life cycle inventory for a dental extraction following the conventional consent process (Scenario A)**

| **Material** | **Product/Process Examples** | **Use per extraction** | **Unit** | **LCI Database Process** | **LCI Database** |
| --- | --- | --- | --- | --- | --- |
| Acrylonitrile | Examination gloves | 12.0 | g | market for acrylonitrile \| acrylonitrile \| Cutoff, S - GLO | ecoinvent 3.9.1 |
| Electricity | Sterilization, laundry processes | 2.39 | kWh | market for electricity, high voltage \| electricity, high voltage \| Cutoff, S - DE |  |
| Electric bicycle | Transport, electric bicycle | 0.34 | km | energy use and operation emissions, electric bicycle \| energy use, electric bicycle \| Cutoff, S - RoW |  |
| Ethanol | Hand disinfection | 0.008 | kg | market for ethanol, without water, in 95% solution state, from fermentation \| ethanol, without water, in 95% solution state, from fermentation \| Cutoff, S - RoW |  |
| Plastic | Hygienic seals | 1.82 | g | injection moulding \| injection moulding \| Cutoff, S - RER |  |
| Isopropanol | Surface disinfection | 0.09 | kg | isopropanol production \| isopropanol \| Cutoff, S - RER |  |
| Gas | Sterilization processes | 0.14 | m3 | market for natural gas, high pressure \| natural gas, high pressure \| Cutoff, S - DE |  |
| Soap | Detergent | 39.80 | g | market for soap \| soap \| Cutoff, S - GLO |  |
| Steam | Laundry processes | 5.4 | kg | market for steam, in chemical industry \| steam, in chemical industry \| Cutoff, S - RER |  |
| Steel | Dental instruments | 9.32 | g | market for steel removed by milling, small parts \| steel removed by milling, small parts \| Cutoff, S - GLO |  |
| Water | Dental unit use | 17.3 | kg | market for tap water \| tap water \| Cutoff, S - Europe without Switzerland |  |
| Polyester | Dental shirt, trousers, coat | 0.12 | g | market for textile, nonwoven polyester \| textile, nonwoven polyester \| Cutoff, S - GLO |  |
| Cotton | Dental shirt, trousers, coat | 0.06 | g | market for textile, woven cotton \| textile, woven cotton \| Cutoff, S - GLO |  |
| Paper | Dental bib, paper towels | 60.83 | g | tissue paper production \| tissue paper \| Cutoff, S - RER |  |
| Large lorry | Large Truck | 0.9 | kg*km | market for transport, freight, lorry 16-32 metric ton, EURO6 \| transport, freight, lorry 16-32 metric ton, EURO6 \| Cutoff, S - RER |  |
| Small lorry | Small Truck | 54.12 | kg*km | market for transport, freight, lorry 3.5-7.5 metric ton, EURO6 \| transport, freight, lorry 3.5-7.5 metric ton, EURO6 \| Cutoff, S - RER |  |
| Sea freight | Ship | 860.76 | kg*km | market for transport, freight, sea, container ship \| transport, freight, sea, container ship \| Cutoff, S - GLO |  |
| Electric car | Passenger transport | 0.45 | km | market for transport, passenger car, electric \| transport, passenger car, electric \| Cutoff, S - GLO |  |
| EURO4 large diesel car | Passenger transport | 0.3 | km | market for transport, passenger car, large size, diesel, EURO 4 \| transport, passenger car, large size, diesel, EURO 4 \| Cutoff, S - GLO |  |
| EURO3 large petrol car | Passenger transport | 0.11 | km | market for transport, passenger car, large size, petrol, EURO 3 \| transport, passenger car, large size, petrol, EURO 3 \| Cutoff, S - GLO |  |
| EURO4 large petrol car | Passenger transport | 0.34 | km | market for transport, passenger car, large size, petrol, EURO 4 \| transport, passenger car, large size, petrol, EURO 4 \| Cutoff, S - GLO |  |
| EURO5 large petrol car | Passenger transport | 0.32 | km | market for transport, passenger car, large size, petrol, EURO 5 \| transport, passenger car, large size, petrol, EURO 5 \| Cutoff, S - GLO |  |
| EURO4 medium diesel car | Passenger transport | 0.74 | km | market for transport, passenger car, medium size, diesel, EURO 4 \| transport, passenger car, medium size, diesel, EURO 4 \| Cutoff, S - GLO |  |
| EURO5 medium diesel car | Passenger transport | 0.47 | km | market for transport, passenger car, medium size, diesel, EURO 5 \| transport, passenger car, medium size, diesel, EURO 5 \| Cutoff, S - GLO |  |
| EURO4 medium gas car | Passenger transport | 6.51 | km | market for transport, passenger car, medium size, natural gas, EURO 4 \| transport, passenger car, medium size, natural gas, EURO 4 \| Cutoff, S - GLO |  |
| EURO4 medium petrol car | Passenger transport | 8.18 | km | market for transport, passenger car, medium size, petrol, EURO 4 \| transport, passenger car, medium size, petrol, EURO 4 \| Cutoff, S - GLO |  |
| EURO5 medium petrol car | Passenger transport | 0.6 | km | market for transport, passenger car, medium size, petrol, EURO 5 \| transport, passenger car, medium size, petrol, EURO 5 \| Cutoff, S - GLO |  |
| EURO3 small petrol car | Passenger transport | 0.02 | km | market for transport, passenger car, small size, petrol, EURO 3 \| transport, passenger car, small size, petrol, EURO 3 \| Cutoff, S - GLO |  |
| EURO4 small petrol car | Passenger transport | 4.51 | km | market for transport, passenger car, small size, petrol, EURO 4 \| transport, passenger car, small size, petrol, EURO 4 \| Cutoff, S - GLO |  |
| EURO5 small petrol car | Passenger transport | 0.02 | km | market for transport, passenger car, small size, petrol, EURO 5 \| transport, passenger car, small size, petrol, EURO 5 \| Cutoff, S - GLO |  |
| coach | Passenger transport | 1.51 | p*km | market for transport, passenger coach \| transport, passenger coach \| Cutoff, S - GLO |  |
| train | Passenger transport | 27.88 | p*km | market for transport, passenger train \| transport, passenger train \| Cutoff, S - GLO |  |
| bicycle | Passenger transport | 3.4 | p*km | market for transport, passenger, bicycle \| transport, passenger, bicycle \| Cutoff, S - GLO |  |
| electric scooter | Passenger transport | 2.56 | km | market for transport, passenger, electric scooter \| transport, passenger, electric scooter \| Cutoff, S - GLO |  |
| motor scooter | Passenger transport | 0.19 | p*km | market for transport, passenger, motor scooter \| transport, passenger, motor scooter \| Cutoff, S - GLO |  |
| tram | Passenger transport | 1.27 | p*km | market for transport, tram \| transport, tram \| Cutoff, S - GLO |  |
| Waste | Waste Treatment | 84.15 | g | market for municipal solid waste \| municipal solid waste \| Cutoff, S - DE |  |
| Wastewater | Dental unit use | 17.3 | l | market for wastewater, average \| wastewater, average \| Cutoff, S - Europe without Switzerland |  |

**Table 16.** Life cycle inventory for a dental extraction following the conventional consent process (Scenario A).

**Life cycle inventory for a dental extraction following a fully digital consent process (Scenario B)**

| **Material** | **Product/Process Examples** | **Use per extraction** | **Unit** | **LCI Database Process** | **LCI Database** |
| --- | --- | --- | --- | --- | --- |
| Acrylonitrile | Examination gloves | 6.0 | g | market for acrylonitrile \| acrylonitrile \| Cutoff, S - GLO | ecoinvent 3.9.1 |
| Electricity | Sterilization, laundry processes | 2.39 | kWh | market for electricity, high voltage \| electricity, high voltage \| Cutoff, S - DE |  |
| Electric bicycle | Transport, electric bicycle | 0.22 | km | market for energy use, electric bicycle \| energy use, electric bicycle \| Cutoff, S - GLO |  |
| Ethanol | Hand desinfection | 0.004 | kg | market for ethanol, without water, in 95% solution state, from fermentation \| ethanol, without water, in 95% solution state, from fermentation \| Cutoff, S - RoW |  |
| Plastic | Hygienic seals | 1.82 | g | injection moulding \| injection moulding \| Cutoff, S - RER |  |
| Isopropanol | Surface disinfection | 0.045 | kg | isopropanol production \| isopropanol \| Cutoff, S - RER |  |
| Gas | Sterilization processes | 0.12 | m3 | market for natural gas, high pressure \| natural gas, high pressure \| Cutoff, S - DE |  |
| Soap | Detergent | 29.8 | g | market for soap \| soap \| Cutoff, S - GLO |  |
| Steam | Laundry processes | 5.4 | kg | market for steam, in chemical industry \| steam, in chemical industry \| Cutoff, S - RER |  |
| Steel | Dental instruments | 9.32 | g | market for steel removed by milling, small parts \| steel removed by milling, small parts \| Cutoff, S - GLO |  |
| Water | Dental unit use | 16.13 | kg | market for tap water \| tap water \| Cutoff, S - Europe without Switzerland |  |
| Polyester | Dental shirt, trousers, coat | 0.10 | g | market for textile, nonwoven polyester \| textile, nonwoven polyester \| Cutoff, S - GLO |  |
| Cotton | Dental shirt, trousers, coat | 0.05 | g | market for textile, woven cotton \| textile, woven cotton \| Cutoff, S - GLO |  |
| Paper | Dental bib, paper towels | 56.83 | g | tissue paper production \| tissue paper \| Cutoff, S - RER |  |
| Large lorry | Large Truck | 0.9 | kg*km | market for transport, freight, lorry 16-32 metric ton, EURO6 \| transport, freight, lorry 16-32 metric ton, EURO6 \| Cutoff, S - RER |  |
| Small lorry | Small Truck | 54.12 | kg*km | market for transport, freight, lorry 3.5-7.5 metric ton, EURO6 \| transport, freight, lorry 3.5-7.5 metric ton, EURO6 \| Cutoff, S - RER |  |
| Sea freight | Ship | 860.76 | kg*km | market for transport, freight, sea, container ship \| transport, freight, sea, container ship \| Cutoff, S - GLO |  |
| Electric car | Passenger transport | 0.22 | km | market for transport, passenger car, electric \| transport, passenger car, electric \| Cutoff, S - GLO |  |
| EURO4 large diesel car | Passenger transport | 0.15 | km | market for transport, passenger car, large size, diesel, EURO 4 \| transport, passenger car, large size, diesel, EURO 4 \| Cutoff, S - GLO |  |
| EURO3 large petrol car | Passenger transport | 0.08 | km | market for transport, passenger car, large size, petrol, EURO 3 \| transport, passenger car, large size, petrol, EURO 3 \| Cutoff, S - GLO |  |
| EURO4 large petrol car | Passenger transport | 0.17 | km | market for transport, passenger car, large size, petrol, EURO 4 \| transport, passenger car, large size, petrol, EURO 4 \| Cutoff, S - GLO |  |
| EURO5 large petrol car | Passenger transport | 0.24 | km | market for transport, passenger car, large size, petrol, EURO 5 \| transport, passenger car, large size, petrol, EURO 5 \| Cutoff, S - GLO |  |
| EURO4 medium diesel car | Passenger transport | 0.47 | km | market for transport, passenger car, medium size, diesel, EURO 4 \| transport, passenger car, medium size, diesel, EURO 4 \| Cutoff, S - GLO |  |
| EURO5 medium diesel car | Passenger transport | 0.23 | km | market for transport, passenger car, medium size, diesel, EURO 5 \| transport, passenger car, medium size, diesel, EURO 5 \| Cutoff, S - GLO |  |
| EURO4 medium gas car | Passenger transport | 3.26 | km | market for transport, passenger car, medium size, natural gas, EURO 4 \| transport, passenger car, medium size, natural gas, EURO 4 \| Cutoff, S - GLO |  |
| EURO4 medium petrol car | Passenger transport | 4.15 | km | market for transport, passenger car, medium size, petrol, EURO 4 \| transport, passenger car, medium size, petrol, EURO 4 \| Cutoff, S - GLO |  |
| EURO5 medium petrol car | Passenger transport | 0.3 | km | market for transport, passenger car, medium size, petrol, EURO 5 \| transport, passenger car, medium size, petrol, EURO 5 \| Cutoff, S - GLO |  |
| EURO3 small petrol car | Passenger transport | 0.02 | km | market for transport, passenger car, small size, petrol, EURO 3 \| transport, passenger car, small size, petrol, EURO 3 \| Cutoff, S - GLO |  |
| EURO4 small petrol car | Passenger transport | 2.61 | km | market for transport, passenger car, small size, petrol, EURO 4 \| transport, passenger car, small size, petrol, EURO 4 \| Cutoff, S - GLO |  |
| EURO5 small petrol car | Passenger transport | 0.02 | km | market for transport, passenger car, small size, petrol, EURO 5 \| transport, passenger car, small size, petrol, EURO 5 \| Cutoff, S - GLO |  |
| coach | Passenger transport | 0.76 | p*km | market for transport, passenger coach \| transport, passenger coach \| Cutoff, S - GLO |  |
| train | Passenger transport | 15.39 | p*km | market for transport, passenger train \| transport, passenger train \| Cutoff, S - GLO |  |
| bicycle | Passenger transport | 1.83 | p*km | market for transport, passenger, bicycle \| transport, passenger, bicycle \| Cutoff, S - GLO |  |
| electric scooter | Passenger transport | 1.31 | km | market for transport, passenger, electric scooter \| transport, passenger, electric scooter \| Cutoff, S - GLO |  |
| motor scooter | Passenger transport | 0.1 | p*km | market for transport, passenger, motor scooter \| transport, passenger, motor scooter \| Cutoff, S - GLO |  |
| tram | Passenger transport | 0.73 | p*km | market for transport, tram \| transport, tram \| Cutoff, S - GLO |  |
| Waste | Waste Treatment | 74.12 | g | market for municipal solid waste \| municipal solid waste \| Cutoff, S - DE |  |
| Wastewater | Dental unit use | 15.97 | l | market for wastewater, average \| wastewater, average \| Cutoff, S - Europe without Switzerland |  |

**Table 17.** Life cycle inventory for a dental extraction following a fully digital consent process (Scenario B)

**Life cycle inventory for a dental extraction without travel (supplementary analysis)**

| **Material** | **Product/Process Examples** | **Use per extraction** | **Unit** | **LCI Database Process** | **LCI Database** |
| --- | --- | --- | --- | --- | --- |
| Acrylonitrile | Examination gloves | 12.0 | g | market for acrylonitrile \| acrylonitrile \| Cutoff, S - GLO | ecoinvent 3.9.1 |
| Electricity | Sterilization, laundry processes | 2.39 | kWh | market for electricity, high voltage \| electricity, high voltage \| Cutoff, S - DE |  |
| Ethanol | Hand desinfection | 0.008 | kg | market for ethanol, without water, in 95% solution state, from fermentation \| ethanol, without water, in 95% solution state, from fermentation \| Cutoff, S - RoW |  |
| Plastic | Hygienic seals | 1.82 | g | injection moulding \| injection moulding \| Cutoff, S - RER |  |
| Isopropanol | Surface disinfection | 0.09 | kg | isopropanol production \| isopropanol \| Cutoff, S - RER |  |
| Gas | Sterilization processes | 0.14 | m3 | market for natural gas, high pressure \| natural gas, high pressure \| Cutoff, S - DE |  |
| Soap | Detergent | 39.8 | g | market for soap \| soap \| Cutoff, S - GLO |  |
| Steam | Laundry processes | 5.4 | kg | market for steam, in chemical industry \| steam, in chemical industry \| Cutoff, S - RER |  |
| Steel | Dental instruments | 9.32 | g | market for steel removed by milling, small parts \| steel removed by milling, small parts \| Cutoff, S - GLO |  |
| Water | Dental unit use | 17.3 | kg | market for tap water \| tap water \| Cutoff, S - Europe without Switzerland |  |
| Polyester | Dental shirt, trousers, coat | 0.12 | g | market for textile, nonwoven polyester \| textile, nonwoven polyester \| Cutoff, S - GLO |  |
| Cotton | Dental shirt, trousers, coat | 0.06 | g | market for textile, woven cotton \| textile, woven cotton \| Cutoff, S - GLO |  |
| Paper | Dental bib, paper towels | 60.83 | g | tissue paper production \| tissue paper \| Cutoff, S - RER |  |
| Large lorry | Large Truck | 0.9 | kg*km | market for transport, freight, lorry 16-32 metric ton, EURO6 \| transport, freight, lorry 16-32 metric ton, EURO6 \| Cutoff, S - RER |  |
| Small lorry | Small Truck | 54.12 | kg*km | market for transport, freight, lorry 3.5-7.5 metric ton, EURO6 \| transport, freight, lorry 3.5-7.5 metric ton, EURO6 \| Cutoff, S - RER |  |
| Sea freight | Ship | 860.76 | kg*km | market for transport, freight, sea, container ship \| transport, freight, sea, container ship \| Cutoff, S - GLO |  |
| Waste | Waste Treatment | 84.15 | g | market for municipal solid waste \| municipal solid waste \| Cutoff, S - DE |  |
| Wastewater | Dental unit use | 17.3 | l | market for wastewater, average \| wastewater, average \| Cutoff, S - Europe without Switzerland |  |

**Table 18.** Life cycle inventory for a dental extraction without travel (supplementary analysis).

For each component of the life cycle inventory, product/process examples, amount of use per extraction, units, the LCI database process and the database (here: ecoinvent version 3.9.1) are listed. Amounts were put into the program using nine decimal digits of accuracy.
