# Supplementary material for: Environmental Impact of a Tooth Extraction: Life Cycle Analysis in a University Hospital Setting
Source: Community Dent Oral Epidemiol. 2025 Jun 27;54(1):30–9. doi: 10.1111/cdoe.70003 (PMC12808852; doi:10.1111/cdoe.70003)
Supplement: Supplementary file 6 — Appendix S6 Supporting Information [file CDOE-54-30-s005.docx]

# Appendix 6. Life cycle impact assessment methods and their specifications.

**Life cycle impact assessment methods**

| **Impact category** | **Indicator** | **Unit** | **Recommended default LCIA model** | **Source of characterization factors** | **Robust-ness** | **Selected method in OpenLCA** |
| --- | --- | --- | --- | --- | --- | --- |
| Acidification | Accumulated Exceedance (AE) | mol H+ eq | Accumulated Exceedance (Seppälä et al. 2006, Posch et al. 2008) | EF - 2017 | II | ILCD 2011 Midpoint+ |
| Climate change | Radiative forcing as Global Warming Potential (GWP100) | kg CO_2_ eq | Baseline model of 100 years of the IPCC (based on IPCC 2013) | EF - 2017 | I | IPCC 2021 |
| Freshwater ecotoxicity | Comparative Toxic Unit for ecosystems (CTU_e_) | CTUe | USEtox model (Rosenbaum et al. 2008) | EF - 2017 | II/III | ILCD 2011 Midpoint+ |
| Freshwater eutrophication | Fraction of nutrients reaching freshwater end compartment | kg P eq | EUTREND model (Struijs et al 2009) as implemented in ReCiPe | EF - 2017 | II | ILCD 2011 Midpoint+ |
| Human toxicity, cancer effects | Comparative Toxic Unit for humans (CTU_h_) | CTUh | USEtox model (Rosenbaum et al. 2008) | EF - 2017 | II/III | ILCD 2011 Midpoint+ |
| Human toxicity, non-cancer effects | Comparative Toxic Unit for humans (CTU_h_) | CTUh | USEtox model (Rosenbaum et al. 2008) | EF - 2017 | II/III | ILCD 2011 Midpoint+ |
| Ionizing radiation HH | Human exposure efficiency relative to U^235^ | kBq U235 eq | Human helath effect model as developed by Dreicer et al. 1995 (Frischknecht et al. 2000) | EF - 2017 | II | ILCD 2011 Midpoint+ |
| Land use | Soil quality index^6^ (Biotic production, Erosion resistance, Mechanical filtration and Groundwater replenishment) | Dimensionless, aggregated index of: kg biotic production/(m^2^*a)^7^ kg soil/(m^2^*a)  m^3^ water/ (m^2^*a) m^3^ g.water/ (m^2^*a) | Soil quality index based on LANCA (Beck et al. 2010 and Bos et al. 2016) | EF - 2017 | III | ILCD 2011 Midpoint+ |
| Marine eutrophication | Fraction of nutrients reaching marine end compartment (N) | kg N eq | EUTREND model (Struijs et al. 2009) as implemented in ReCiPe | EF - 2017 | II | ILCD 2011 Midpoint+ |
| Resource use, minerals and metals | Abiotic resource depletion (ADP ultimate reserves) | kg Sb eq | CML Guinée et al. 2002 and van Oers et al. 2002 | EF - 2017 | III | CML v4.8 2016 |
| Resource use, energy carriers | Abiotic resource depletion – fossil fuels (ADP fossil)^8^ | MJ | CML Guinée et al. 2002 and van Oers et al. 2002 | EF - 2017 | III | CML v4.8 2016 |
| Ozone depletion | Ozone Depletion Potential (ODP) | kg CFC-11 eq | Steady-state ODPs as in WMO 1999 | EF - 2017 | I | ILCD 2011 Midpoint+ |
| Particulate matter/Respiratory inorganics | Human health effects associated with exposure to PM_2.5_ | kg PM2.5 eq | PM model recommended by UNEP (UNEP 2016) | EF - 2017 | I | ILCD 2011 Midpoint+ |
| Photochemical ozone formation | Tropospheric ozone concentration increase | kg NMVOC eq | LOTOS-EUROS (Van Zelm et al. 2008) as applied in ReCiPe 2008 | EF - 2017 | II | ILCD 2011 Midpoint+ |
| Terrestrial eutrophication | Accumulated exceedance (AE) | mol N eq | Accumulated Exceedance (Seppälä et al. 2006, Posch et al. 2008) | EF - 2017 | II | ILCD 2011 Midpoint+ |
| Water resource depletion | User deprivation potential (deprivation-weighted water consumption) | kg world eq. deprived | Available WAter REmaining (AWARE) in UNEP, 2016 | EF - 2017 | III | ILCD 2011 Midpoint+ |

**Table 19.** Life cycle impact assessment methods.
