# Supplementary material for: Environmental Impact of a Tooth Extraction: Life Cycle Analysis in a University Hospital Setting
Source: Community Dent Oral Epidemiol. 2025 Jun 27;54(1):30–9. doi: 10.1111/cdoe.70003 (PMC12808852; doi:10.1111/cdoe.70003)
Supplement: Supplementary file 7 — Appendix S7 Supporting Information [file CDOE-54-30-s001.docx]

# Appendix 7: Life cyle impact assessment results for dental extractions with conventional and digital consent processes and a supplementary analysis.

**Life cycle impact assessment results for one dental extraction with dental travel (conventional consent process, Scenario A)**

| **Impact Category** | **Unit** | **Plastic** | **Isopropanol** | **Acrylonitrile** | **Electricity** | **Electric bicycle** | **Ethanol** | **Waste** | **Gas** |
| --- | --- | --- | --- | --- | --- | --- | --- | --- | --- |
| **Acidification** | molc H+ eq | 6.71E-06 | 0.000767 | 0.000263 | 0.002384 | 1.29E-05 | 8.68E-05 | 2.9E-05 | 0.000121 |
| **Climate change** | kg CO_2_ eq | 0.001634 | 0.176419 | 0.043552 | 1.137786 | 0.002493 | 0.00691 | 0.044719 | 0.080236 |
| **Freshwater ecotoxicity** | CTUe | 0.016511 | 1.352779 | 0.291939 | 14.159527 | 0.049338 | 0.269501 | 6.180475 | 0.286248 |
| **Freshwater eutrophication** | kg P eq | 9.25E-07 | 1.94E-05 | 6.35E-06 | 0.001638 | 1.18E-06 | 2.08E-06 | 3.29E-06 | 4.08E-06 |
| **Human toxicity, cancer effects** | CTUh | 1.38E-10 | 7.16E-09 | 1.73E-09 | 1.34E-07 | 1.53E-10 | 4.49E-10 | 4.02E-09 | 7.27E-09 |
| **Human toxicity, non-cancer effects** | CTUh | 3.7E-10 | 2.33E-08 | 4.75E-09 | 3.96E-07 | 6.48E-10 | 1.09E-08 | 6.99E-08 | 3.35E-09 |
| **Ionizing radiation** | kg Sb-Eq | 0.000625 | 0.0073 | 0.000552 | 0.271567 | 0.000353 | 0.000111 | 8.47E-05 | 0.000577 |
| **Land use** | kBq U235 eq | 0.001737 | 0.049222 | 0.015498 | 0.42923 | 0.001805 | 0.162287 | 0.006603 | 0.041686 |
| **Marine eutrophication** | kg C deficit | 1.24E-06 | 9.63E-05 | 0.000155 | 0.000804 | 2.46E-06 | 3.71E-05 | 1.6E-05 | 4.1E-05 |
| **Resource use, energy carriers** | MJ | 0.025297 | 5.079802 | 0.920338 | 12.209598 | 0.026953 | 0.043263 | 0.039186 | 5.714346 |
| **Resource use, minerals and metals** | kg N eq | 3.77E-09 | 8.03E-07 | 1.65E-07 | 9.39E-07 | 1.38E-08 | 6.21E-08 | 8.08E-09 | 4.92E-08 |
| **Ozone depletion** | kg CFC-11 eq | 5.84E-11 | 1.74E-09 | 3.14E-10 | 7.96E-09 | 1.49E-11 | 4.74E-10 | 1.35E-10 | 1.31E-08 |
| **Particulate matter/Respira-tory inorganics** | kg PM2.5 eq | 5.51E-07 | 7.09E-05 | 2.06E-05 | 0.000150 | 2.35E-06 | 5.26E-06 | 2.66E-06 | 1.05E-05 |
| **Photochemical ozone formation** | kg NMVOC eq | 4.87E-06 | 0.000742 | 0.000109 | 0.001543 | 7.29E-06 | 3.14E-05 | 3.58E-05 | 0.000504 |
| **Terrestrial eutrophication** | molc N eq | 1.15E-05 | 0.001025 | 0.000812 | 0.005787 | 2.47E-05 | 0.000362 | 0.000132 | 0.000439 |
| **Water resource depletion** | m3 water eq | -5.18E-05 | -0.002291 | -0.000277 | 0.000219 | -7.95E-06 | 0.000192 | -3.15E-05 | 8.71E-06 |

| **Soap** | **Steam** | **Steel** | **Water** | **Polyester** | **Cotton** | **Large lorry** | **Small lorry** | **Sea freight** | **Electric car** |
| --- | --- | --- | --- | --- | --- | --- | --- | --- | --- |
| 0.000860 | 0.004511 | 0.000224 | 2.91E-05 | 3.48E-06 | 8.57E-06 | 3.63E-07 | 6.39E-05 | 0.000260 | 0.00062 |
| 0.208997 | 1.63981 | 0.050697 | 0.005343 | 0.000675 | 0.000697 | 0.000166 | 0.030134 | 0.008748 | 0.103866 |
| 2.657114 | 3.340229 | 2.072477 | 0.136999 | 0.008774 | 0.021834 | 0.001513 | 0.289908 | 0.030509 | 5.048105 |
| 0.001378 | 0.000185 | 2.27E-05 | 3.54E-06 | 1.82E-07 | 4.96E-07 | 1.19E-08 | 2.56E-06 | 2.96E-07 | 5.26E-05 |
| 9.96E-09 | 4.3E-08 | 4.27E-08 | 6.41E-09 | 6.64E-11 | 7.28E-11 | 1.21E-11 | 2.61E-09 | 5.16E-10 | 1.75E-08 |
| 5.96E-08 | 1.14E-07 | 2.51E-08 | 3.56E-09 | 1.55E-10 | 2.69E-10 | 3.84E-11 | 6.81E-09 | 4.26E-10 | 4.97E-08 |
| 0.004169 | 0.040230 | 0.004186 | 0.00202 | 4.03E-05 | 4.43E-05 | 3.2E-06 | 0.000843 | 5.14E-05 | 0.012192 |
| 7.048171 | 0.586450 | 0.144527 | 0.005813 | 0.000628 | 0.006444 | 0.000314 | 0.044483 | 0.006744 | 0.136556 |
| 0.001024 | 0.000752 | 6.48E-05 | 5.72E-06 | 7.72E-07 | 1.18E-05 | 9.23E-08 | 1.5E-05 | 6.51E-05 | 0.000118 |
| 0.714215 | 22.102623 | 0.481886 | 0.061347 | 0.011740 | 0.006273 | 0.002327 | 0.412321 | 0.107037 | 1.192044 |
| 7.76E-07 | 5.32E-07 | 2.87E-07 | 2.80E-08 | 5.20E-09 | 3.19E-09 | 5.44E-10 | 1.31E-07 | 8.45E-09 | 2.97E-06 |
| 4.12E-09 | 4.29E-08 | 9.21E-10 | 1.50E-10 | 1.27E-09 | 6.13E-12 | 2.97E-12 | 5.40E-10 | 1.08E-10 | 1.57E-09 |
| 0.000165 | 0.000498 | 4.92E-05 | 3.43E-06 | 4.71E-07 | 5.57E-07 | 6.12E-08 | 8.9E-06 | 9.53E-06 | 0.000113 |
| 0.000519 | 0.003771 | 0.000174 | 1.88E-05 | 3.18E-06 | 2.72E-06 | 5.52E-07 | 9.25E-05 | 0.000194 | 0.000571 |
| 0.003187 | 0.007747 | 0.000512 | 5.44E-05 | 8.69E-06 | 3.28E-05 | 9.32E-07 | 0.000150 | 0.00072 | 0.001133 |
| 0.002321 | -0.06590 | -7.44E-05 | 0.002783 | -2.94E-06 | 6.14E-05 | -1.33E-07 | -3.87E-05 | -1.54E-06 | -0.000295 |

| **Passenger coach** | **Passenger train** | **Bicycle** | **Electric scooter** | **Motor scooter** | **Tram** | **Tissue paper** | **Wastewater** | **EURO 3 small petrol car** |
| --- | --- | --- | --- | --- | --- | --- | --- | --- |
| 0.000657 | 0.011723 | 0.000234 | 0.000878 | 8.86E-05 | 0.000582 | 0.000458 | 3.13E-05 | 1.74E-05 |
| 0.090863 | 2.071001 | 0.040086 | 0.137461 | 0.025364 | 0.111359 | 0.097475 | 0.006292 | 0.006494 |
| 0.529729 | 17.859754 | 1.911677 | 7.715317 | 0.217385 | 1.304491 | 1.217967 | 0.217466 | 0.166528 |
| 6.83E-06 | 0.000818 | 1.27E-05 | 6.34E-05 | 2.60E-06 | 4.67E-05 | 8.50E-05 | 2.96E-05 | 7.36E-07 |
| 4.46E-09 | 2.05E-07 | 1.81E-08 | 2.45E-08 | 1.37E-09 | 1.51E-08 | 7.74E-09 | 1.62E-09 | 6.77E-10 |
| 7.74E-09 | 3.89E-07 | 3.26E-08 | 8.26E-08 | 4.57E-09 | 2.44E-08 | 1.48E-07 | 5.46E-08 | 1.26E-09 |
| 0.001366 | 0.259383 | 0.001573 | 0.013127 | 0.000553 | 0.013633 | 0.01387 | 0.001005 | 0.000127 |
| 0.164531 | 2.662885 | 0.063649 | 0.191988 | 0.021924 | 0.102943 | 2.228261 | 0.010677 | 0.007758 |
| 0.000287 | 0.003199 | 4.52E-05 | 0.000161 | 2.59E-05 | 0.000134 | 0.000155 | 0.000344 | 3.51E-06 |
| 1.250791 | 23.002361 | 0.453959 | 1.644246 | 0.30712 | 1.202851 | 1.125677 | 0.053708 | 0.081319 |
| 1.43E-07 | 3.95E-06 | 1.92E-07 | 3.89E-06 | 5.92E-08 | 3.68E-07 | 3.22E-07 | 3.10E-08 | 5.51E-08 |
| 1.32E-09 | 1.49E-08 | 4.6E-10 | 2.03E-09 | 3.35E-10 | 7.64E-10 | 3.55E-09 | 7.09E-11 | 1.24E-10 |
| 4.36E-05 | 0.001762 | 4.22E-05 | 0.000138 | 1.13E-05 | 0.000102 | 4.94E-05 | 3.43E-06 | 2.66E-06 |
| 0.00091 | 0.009734 | 0.000152 | 0.000532 | 0.000569 | 0.000603 | 0.000278 | 2.10E-05 | 2.14E-05 |
| 0.003095 | 0.033276 | 0.000448 | 0.001481 | 0.000274 | 0.00136 | 0.001091 | 9.87E-05 | 3.57E-05 |
| -4.28E-05 | -0.005916 | -6.91E-05 | -0.000339 | -1.51E-05 | -0.000299 | -0.002457 | -4.36E-05 | -4.06E-06 |

| **EURO 3 large petrol car** | **EURO 4 small petrol car** | **EURO 4 medium petrol car** | **EURO 4 medium diesel car** | **EURO 4 medium gas car** | **EURO 4 large petrol car** | **EURO 4 large diesel car** | **EURO 5 small petrol car** | **EURO 5 medium petrol car** | **EURO 5 medium diesel car** | **EURO 5 large petrol car** |
| --- | --- | --- | --- | --- | --- | --- | --- | --- | --- | --- |
| 0.000145 | 0.0037 | 0.008539 | 0.001021 | 0.005196 | 0.000431 | 0.000485 | 1.59E-05 | 0.000611 | 0.000692 | 0.000397 |
| 0.052093 | 1.380937 | 3.116687 | 0.252708 | 2.018688 | 0.154968 | 0.127398 | 0.005851 | 0.220178 | 0.156998 | 0.141234 |
| 1.516609 | 37.478526 | 90.314785 | 8.348236 | 71.448913 | 4.682385 | 4.226589 | 0.165957 | 6.616994 | 5.29876 | 4.402807 |
| 6.51E-06 | 0.000165 | 0.0003906 | 3.65E-05 | 0.000303 | 2.0E-05 | 1.84E-05 | 7.27E-07 | 2.85E-05 | 2.31E-05 | 1.88E-05 |
| 6.10E-09 | 1.52E-07 | 3.64E-07 | 3.28E-08 | 2.94E-07 | 1.88E-08 | 1.66E-08 | 6.7E-10 | 2.66E-08 | 2.1E-08 | 1.76E-08 |
| 1.12E-08 | 2.81E-07 | 6.7E-07 | 6.22E-08 | 5.06E-07 | 3.45E-08 | 3.14E-08 | 1.24E-09 | 4.89E-08 | 3.93E-08 | 3.24E-08 |
| 0.001083 | 0.028121 | 0.065413 | 0.005515 | 0.046625 | 0.003318 | 0.002752 | 0.000123 | 0.004756 | 0.00349 | 0.003099 |
| 0.065575 | 1.697798 | 3.933147 | 0.358351 | 2.185901 | 0.198958 | 0.180258 | 0.007357 | 0.283189 | 0.22501 | 0.184347 |
| 2.85E-05 | 0.000717 | 0.00164 | 0.000293 | 0.001036 | 8.23E-05 | 0.000132 | 3.05E-06 | 0.000116 | 0.00021 | 7.51E-05 |
| 0.653004 | 17.316125 | 39.098586 | 3.339723 | 29.889404 | 1.94464 | 1.683878 | 0.073388 | 2.762938 | 2.074078 | 1.772757 |
| 5.03E-07 | 1.24E-05 | 2.99E-05 | 2.66E-06 | 2.38E-05 | 1.55E-06 | 1.35E-06 | 5.5E-08 | 2.2E-06 | 1.69E-06 | 1.46E-06 |
| 1.02E-09 | 2.67E-08 | 6.11E-08 | 4.5E-09 | 4.88E-08 | 3.07E-09 | 2.27E-09 | 1.14E-10 | 4.35E-09 | 2.82E-09 | 2.81E-09 |
| 2.29E-05 | 0.000589 | 0.001376 | 0.000214 | 0.000968 | 7.0E-05 | 9.8E-05 | 2.58E-06 | 0.000100 | 8.01E-05 | 6.54E-05 |
| 0.000169 | 0.004496 | 0.010076 | 0.001163 | 0.00595 | 0.000499 | 0.000547 | 1.92E-05 | 0.000716 | 0.000796 | 0.000456 |
| 0.000289 | 0.007257 | 0.016599 | 0.003090 | 0.011858 | 0.000833 | 0.001389 | 3.08E-05 | 0.001174 | 0.002227 | 0.00076 |
| -3.48E-05 | -0.000897 | -0.00209 | -0.000176 | -0.068686 | -0.000106 | -8.81E-05 | -3.92E-06 | -0.000151 | -0.000111 | -9.88E-05 |

**Table 20.** Life cycle impact assessment results for one dental extraction with dental travel (conventional consent process, Scenario A).

**Scenario A. Figurative representation of Life Cycle Impact Assessment results for one dental extraction with dental travel (conventional consent process)**

**
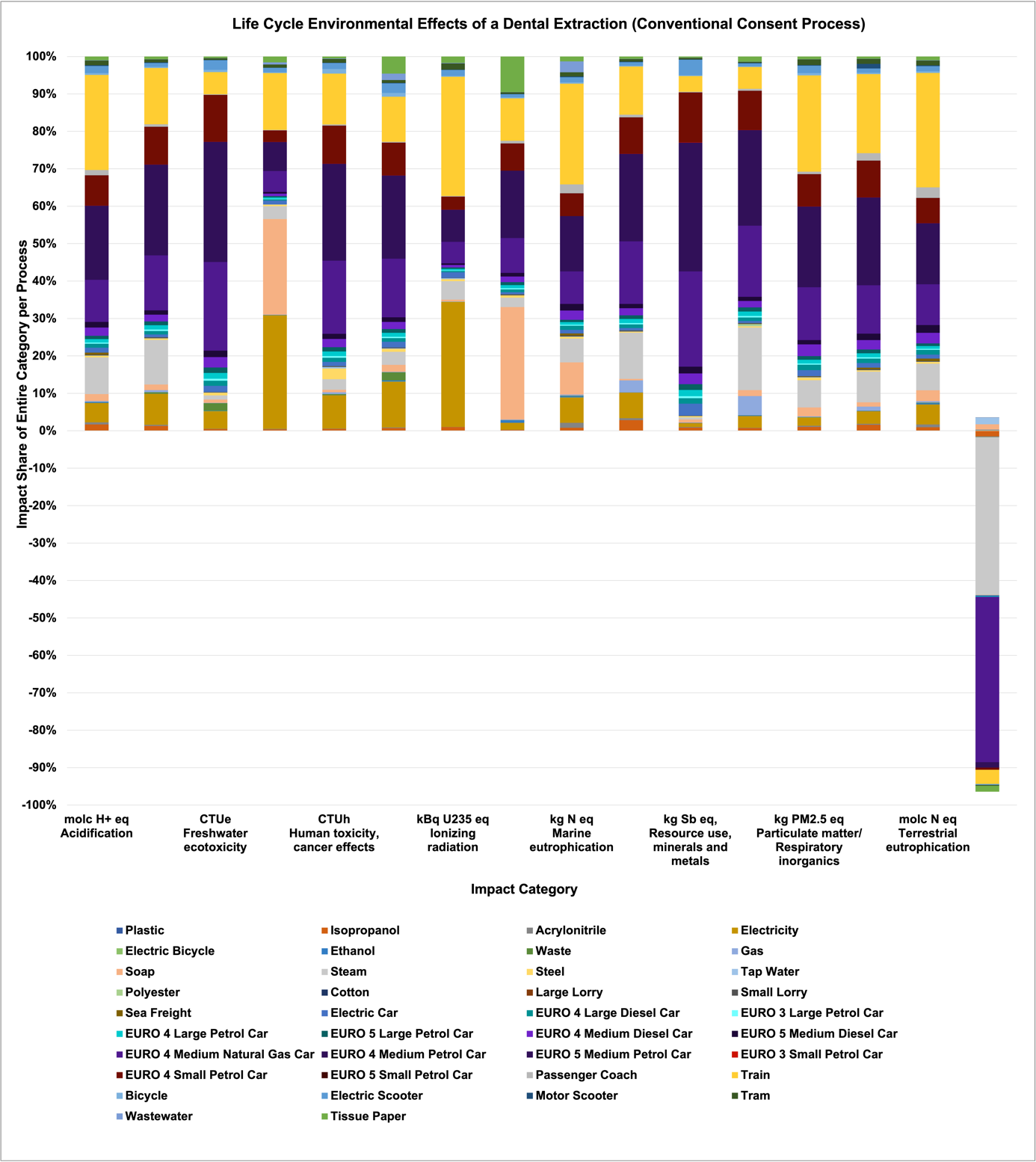
**

**Figure 5.** Scenario A. Life cycle impact assessment results for the entire dental extraction using a conventional medical informed consent process.

Total potential impact and normalized results for a dental extraction following a conventional consent process (scenario A).

| **Impact Category** | **Unit** | **Total potential impact** | **Normalized Result** |
| --- | --- | --- | --- |
| Acidification | molc H+ eq | 0.046152517 | 0.000822682 |
| Climate change | kg CO_2_ eq | 13.75701961 | 0.001822122 |
| Freshwater ecotoxicity | CTUe | 301.8546573 | 0.080709898 |
| Freshwater eutrophication | kg P eq | 0.005398527 | 0.000825462 |
| Human toxicity, cancer effects | CTUh | 1.50581E-06 | 0.121436386 |
| Human toxicity, non-cancer effects | CTUh | 3.23173E-06 | 0.020849818 |
| Ionizing radiation | kBq U235 eq | 0.813880571 | 0.0033771 |
| Land use | kg C deficit | 23.46270649 | 4.51207E-06 |
| Marine eutrophication | kg N eq | 0.011899068 | 0.000391416 |
| Resource use, energy carriers | MJ | 178.8811461 | 0.002752018 |
| Resource use, minerals and metals | kg Sb eq | 9.34267E-05 | 0.001468974 |
| Ozone depletion | kg CFC-11 eq | 2.56402E-07 | 2.10165E-05 |
| Particulate matter/ Respiratory inorganics | kg PM2.5 eq | 0.006851426 | 0.001351368 |
| Photochemical ozone formation | kg NMVOC eq | 0.046032654 | 0.001016171 |
| Terrestrial eutrophication | molc N eq | 0.108806785 | 0.000663456 |
| Water resource depletion | m3 water eq | -0.14501687 | -0.002104746 |

**Table 21.** Total potential impact and normalized results for a dental extraction following a conventional consent process (scenario A).

The total potential burden a dental extraction has in a university clinic/medical care center. Total impacts were normalized using the average expenditure of a person per year using normalization factors from ILCD 2011 Midpoint+ for most data points and EF3.1 factors for those impact categories retrieved from impact assessment methods IPCC 2021 and CML v4.8 2016. The value 1 would represent the total consumption per year^1^

**Life cycle impact assessment results for one dental extraction with dental travel (digital consent process, Scenario B)**

| **Impact Category** | **Unit** | **Plastic** | **Isopropanol** | **Acrylonitrile** | **Electricity** | **Electric bicycle** | **Ethanol** | **Waste** | **Gas** |
| --- | --- | --- | --- | --- | --- | --- | --- | --- | --- |
| **Acidification** | molc H+ eq | 6.71E-06 | 0.000383 | 0.000132 | 0.002384 | 8.35E-06 | 4.34E-05 | 2.55E-05 | 0.000103 |
| **Climate change** | kg CO_2_ eq | 0.001634 | 0.088209 | 0.021776 | 1.137786 | 0.001613 | 0.003456 | 0.039388 | 0.068774 |
| **Freshwater ecotoxicity** | CTUe | 0.016511 | 0.676389 | 0.145969 | 14.159527 | 0.031925 | 0.13475 | 5.443813 | 0.245355 |
| **Freshwater eutrophication** | kg P eq | 9.25E-07 | 9.7E-06 | 3.17E-06 | 0.001638 | 7.66E-07 | 1.04E-06 | 2.9E-06 | 3.49E-06 |
| **Human toxicity, cancer effects** | CTUh | 1.38E-10 | 3.58E-09 | 8.65E-10 | 1.34E-07 | 9.92E-11 | 2.24E-10 | 3.54E-09 | 6.23E-09 |
| **Human toxicity, non-cancer effects** | CTUh | 3.7E-10 | 1.16E-08 | 2.38E-09 | 3.96E-07 | 4.19E-10 | 5.46E-09 | 6.15E-08 | 2.87E-09 |
| **Ionizing radiation** | kg Sb-Eq | 0.000625 | 0.003650 | 0.000276 | 0.271567 | 0.000229 | 5.53E-05 | 7.46E-05 | 0.000494 |
| **Land use** | kBq U235 eq | 0.001737 | 0.024611 | 0.007749 | 0.42923 | 0.001168 | 0.081144 | 0.005816 | 0.035731 |
| **Marine eutrophication** | kg C deficit | 1.24E-06 | 4.82E-05 | 7.77E-05 | 0.000804 | 1.59E-06 | 1.85E-05 | 1.41E-05 | 3.52E-05 |
| **Resource use, energy carriers** | kg N eq | 0.025297 | 2.539901 | 0.460169 | 12.209598 | 0.01744 | 0.021632 | 0.034515 | 4.898011 |
| **Resource use, minerals and metals** | MJ | 3.77E-09 | 4.02E-07 | 8.25E-08 | 9.39E-07 | 8.96E-09 | 3.1E-08 | 7.12E-09 | 4.22E-08 |
| **Ozone depletion** | kg CFC-11 eq | 5.84E-11 | 8.72E-10 | 1.57E-10 | 7.96E-09 | 9.61E-12 | 2.37E-10 | 1.19E-10 | 1.12E-08 |
| **Particulate matter/Respiratory inorganics** | kg PM2.5 eq | 5.51E-07 | 3.54E-05 | 1.03E-05 | 0.000150 | 1.52E-06 | 2.63E-06 | 2.34E-06 | 9.0E-06 |
| **Photochemical ozone formation** | kg NMVOC eq | 4.87E-06 | 0.000371 | 5.47E-05 | 0.001543 | 4.72E-06 | 1.57E-05 | 3.15E-05 | 0.000432 |
| **Terrestrial eutrophication** | molc N eq | 1.15E-05 | 0.000512 | 0.000406 | 0.005787 | 1.6E-05 | 0.000181 | 0.000116 | 0.000377 |
| **Water resource depletion** | m3 water eq | -5.18E-05 | -0.001146 | -0.000138 | 0.000219 | -5.1E-06 | 9.6E-05 | -2.77E-05 | 7.46E-06 |

| **Soap** | **Steam** | **Steel** | **Water** | **Polyester** | **Cotton** | **Large lorry** | **Small Lorry** | **Sea freight** | **Electric car** |
| --- | --- | --- | --- | --- | --- | --- | --- | --- | --- |
| 0.000644 | 0.004511 | 0.000224 | 2.71E-05 | 2.9E-06 | 7.14E-06 | 3.63E-07 | 6.39E-05 | 0.000260 | 0.000303 |
| 0.156485 | 1.63981 | 0.050697 | 0.004982 | 0.000563 | 0.000581 | 0.000166 | 0.030134 | 0.008748 | 0.050779 |
| 1.989497 | 3.340229 | 2.072477 | 0.127733 | 0.007312 | 0.018195 | 0.001513 | 0.289908 | 0.030509 | 2.467963 |
| 0.001032 | 0.000185 | 2.27E-05 | 3.3E-06 | 1.51E-07 | 4.13E-07 | 1.19E-08 | 2.56E-06 | 2.96E-07 | 2.57E-05 |
| 7.46E-09 | 4.3E-08 | 4.27E-08 | 5.98E-09 | 5.54E-11 | 6.07E-11 | 1.21E-11 | 2.61E-09 | 5.16E-10 | 8.54E-09 |
| 4.46E-08 | 1.14E-07 | 2.51E-08 | 3.32E-09 | 1.29E-10 | 2.24E-10 | 3.84E-11 | 6.81E-09 | 4.26E-10 | 2.43E-08 |
| 0.003121 | 0.040230 | 0.004186 | 0.001883 | 3.36E-05 | 3.69E-05 | 3.2E-06 | 0.000843 | 5.14E-05 | 0.00596 |
| 5.277274 | 0.586450 | 0.144527 | 0.00542 | 0.000523 | 0.005370 | 0.000314 | 0.044483 | 0.006744 | 0.066761 |
| 0.000767 | 0.000752 | 6.48E-05 | 5.33E-06 | 6.43E-07 | 9.82E-06 | 9.23E-08 | 1.5E-05 | 6.51E-05 | 5.76E-05 |
| 0.534764 | 22.102623 | 0.481886 | 0.057198 | 0.009784 | 0.005227 | 0.002327 | 0.412321 | 0.107037 | 0.582777 |
| 5.81E-07 | 5.32E-07 | 2.87E-07 | 2.61E-08 | 4.33E-09 | 2.66E-09 | 5.44E-10 | 1.31E-07 | 8.45E-09 | 1.45E-06 |
| 3.09E-09 | 4.29E-08 | 9.21E-10 | 1.4E-10 | 1.06E-09 | 5.11E-12 | 2.97E-12 | 5.40E-10 | 1.08E-10 | 7.66E-10 |
| 0.000123 | 0.000498 | 4.92E-05 | 3.19E-06 | 3.92E-07 | 4.65E-07 | 6.12E-08 | 8.9E-06 | 9.53E-06 | 5.5E-05 |
| 0.000389 | 0.003771 | 0.000174 | 1.76E-05 | 2.65E-06 | 2.27E-06 | 5.52E-07 | 9.25E-05 | 0.000194 | 0.000279 |
| 0.002386 | 0.007747 | 0.000512 | 5.07E-05 | 7.24E-06 | 2.73E-05 | 9.32E-07 | 0.000150 | 0.00072 | 0.000554 |
| 0.001738 | -0.06590 | -7.44E-05 | 0.002595 | -2.45E-06 | 5.12E-05 | -1.33E-07 | -3.87E-05 | -1.54E-06 | -0.00014 |

| **Passenger coach** | **Passenger train** | **Bicycle** | **Electric scooter** | **Motor scooter** | **Tram** | **Tissue paper** | **Wastewater** | **EURO 3 small petrol car** |
| --- | --- | --- | --- | --- | --- | --- | --- | --- |
| 0.000331 | 0.006471 | 0.000126 | 0.000449 | 4.66E-05 | 0.000334 | 0.000427 | 2.68E-05 | 1.74E-05 |
| 0.045732 | 1.143211 | 0.021576 | 0.070341 | 0.013349 | 0.064009 | 0.091066 | 0.0051 | 0.006494 |
| 0.266619 | 9.858738 | 1.028932 | 3.948073 | 0.114413 | 0.749825 | 1.13788 | 0.171743 | 0.166528 |
| 3.44E-06 | 0.000452 | 6.85E-06 | 3.25E-05 | 1.37E-06 | 2.68E-05 | 7.94E-05 | 2.06E-05 | 7.36E-07 |
| 2.25E-09 | 1.13E-07 | 9.73E-09 | 1.25E-08 | 7.23E-10 | 8.7E-09 | 7.23E-09 | 8.27E-10 | 6.77E-10 |
| 3.9E-09 | 2.15E-07 | 1.75E-08 | 4.23E-08 | 2.41E-09 | 1.4E-08 | 1.38E-07 | 5.68E-08 | 1.26E-09 |
| 0.000687 | 0.143182 | 0.000847 | 0.006717 | 0.000291 | 0.007836 | 0.012958 | 0.001008 | 0.000127 |
| 0.082810 | 1.469936 | 0.034258 | 0.098244 | 0.011539 | 0.059172 | 2.081738 | 0.008232 | 0.007758 |
| 0.000144 | 0.001766 | 2.43E-05 | 8.24E-05 | 1.36E-05 | 7.68E-05 | 0.000145 | 0.00029 | 3.51E-06 |
| 0.629537 | 12.697501 | 0.244337 | 0.841391 | 0.161642 | 0.691402 | 1.051656 | 0.038637 | 0.081319 |
| 7.19E-08 | 2.18E-06 | 1.03E-07 | 1.99E-06 | 3.11E-08 | 2.12E-07 | 3.01E-07 | 2.41E-08 | 5.51E-08 |
| 6.66E-10 | 8.25E-09 | 2.47E-10 | 1.04E-09 | 1.76E-10 | 4.39E-10 | 3.32E-09 | 4.83E-11 | 1.24E-10 |
| 2.2E-05 | 0.000973 | 2.27E-05 | 7.07E-05 | 5.95E-06 | 5.86E-05 | 4.62E-05 | 2.07E-06 | 2.66E-06 |
| 0.000458 | 0.005373 | 8.19E-05 | 0.000272 | 0.0003 | 0.000347 | 0.000259 | 1.65E-05 | 2.14E-05 |
| 0.001558 | 0.018369 | 0.000241 | 0.000758 | 0.000144 | 0.000782 | 0.001019 | 8.76E-05 | 3.57E-05 |
| -2.15E-05 | -0.003266 | -3.72E-05 | -0.000173 | -7.97E-06 | -0.000172 | -0.002295 | -4.6E-05 | -4.06E-06 |

| **EURO 3 large petrol car** | **EURO 4 small petrol car** | **EURO 4 medium petrol car** | **EURO 4 medium diesel car** | **EURO 4 medium gas car** | **EURO 4**  **large petrol**  **car** | **EURO 4 large diesel car** | **EURO 5 small petrol car** | **EURO 5 medium petrol car** | **EURO 5 medium diesel car** | **EURO 5 large petrol car** |
| --- | --- | --- | --- | --- | --- | --- | --- | --- | --- | --- |
| 0.000105 | 0.002142 | 0.004332 | 0.000649 | 0.002602 | 0.000215 | 0.000243 | 1.59E-05 | 0.000305 | 0.000338 | 0.000298 |
| 0.037886 | 0.799167 | 1.581204 | 0.160504 | 1.010895 | 0.077484 | 0.063699 | 0.005851 | 0.110089 | 0.076829 | 0.105925 |
| 1.102988 | 21.689347 | 45.819848 | 5.302258 | 35.779333 | 2.341192 | 2.113295 | 0.165957 | 3.308497 | 2.59301 | 3.302105 |
| 4.74E-06 | 9.54E-05 | 0.000198 | 2.32E-05 | 0.000152 | 1.0E-05 | 9.19E-06 | 7.27E-07 | 1.43E-05 | 1.13E-05 | 1.41E-05 |
| 4.44E-09 | 8.78E-08 | 1.85E-07 | 2.08E-08 | 1.47E-07 | 9.4E-09 | 8.29E-09 | 6.7E-10 | 1.33E-08 | 1.02E-08 | 1.32E-08 |
| 8.17E-09 | 1.63E-07 | 3.4E-07 | 3.95E-08 | 2.53E-07 | 1.73E-08 | 1.57E-08 | 1.24E-09 | 2.45E-08 | 1.92E-08 | 2.43E-08 |
| 0.000788 | 0.016274 | 0.033186 | 0.003503 | 0.023348 | 0.001659 | 0.001376 | 0.000123 | 0.002378 | 0.001708 | 0.002325 |
| 0.047691 | 0.98254 | 1.995423 | 0.227601 | 1.094629 | 0.099479 | 0.090129 | 0.007357 | 0.141595 | 0.110111 | 0.138261 |
| 2.07E-05 | 0.000415 | 0.000832 | 0.000186 | 0.000519 | 4.11E-05 | 6.62E-05 | 3.05E-06 | 5.81E-05 | 0.000103 | 5.63E-05 |
| 0.474912 | 10.021083 | 19.83608 | 2.121175 | 14.967659 | 0.97232 | 0.841939 | 0.073388 | 1.381469 | 1.014974 | 1.329567 |
| 3.66E-07 | 7.18E-06 | 1.52E-05 | 1.69E-06 | 1.19E-05 | 7.77E-07 | 6.73E-07 | 5.5E-08 | 1.1E-06 | 8.26E-07 | 1.1E-06 |
| 7.44E-10 | 1.55E-08 | 3.1E-08 | 2.86E-09 | 2.44E-08 | 1.53E-09 | 1.14E-09 | 1.14E-10 | 2.17E-09 | 1.38E-09 | 2.11E-09 |
| 1.67E-05 | 0.000341 | 0.000698 | 0.000136 | 0.000485 | 3.5E-05 | 4.9E-05 | 2.58E-06 | 5.0E-05 | 3.92E-05 | 4.91E-05 |
| 0.000123 | 0.002602 | 0.005112 | 0.000739 | 0.002979 | 0.000249 | 0.000274 | 1.92E-05 | 0.0003578 | 0.000389 | 0.000342 |
| 0.000211 | 0.0042 | 0.008421 | 0.001963 | 0.005938 | 0.000416 | 0.000694 | 3.08E-05 | 0.000587 | 0.001091 | 0.00057 |
| -2.53E-05 | -0.000519 | -0.001060 | -0.000112 | -0.034396 | -5.3E-05 | -4.4E-05 | -3.92E-06 | -7.57E-05 | -5.43E-05 | -7.41E-05 |

**Table 22.** Life cycle impact assessment results for one dental extraction with dental travel (digital consent process, Scenario B)

**Scenario B. Figurative representation of Life Cycle Impact Assessment results for one dental extraction with dental travel (digital consent process)**


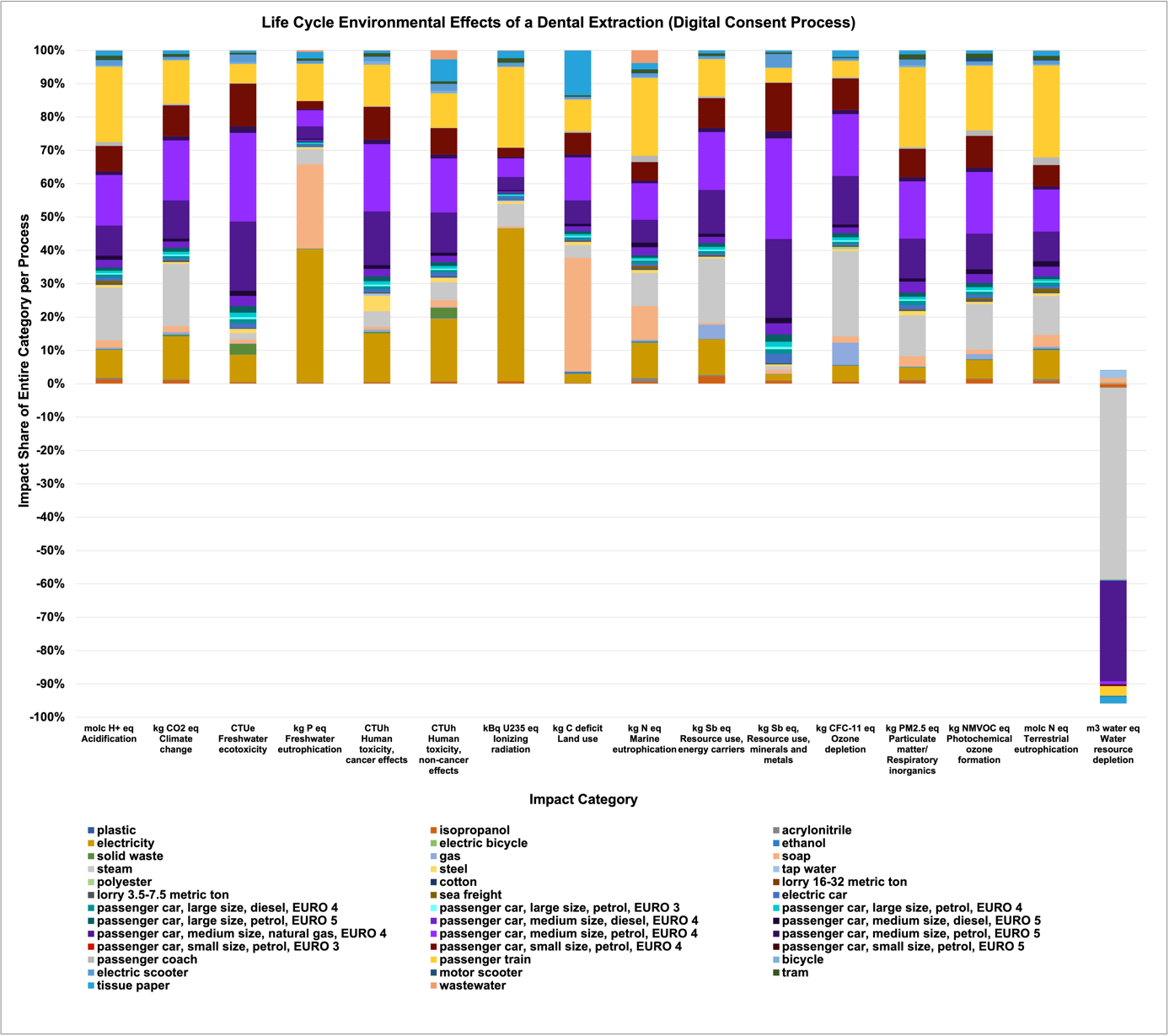


**Figure 6.** Scenario B. Life cycle impact assessment results for the entire dental extraction using a digital medical informed consent process.

Total potential impact and normalized results for a dental extraction following a digital consent process (scenario B).

| **Impact Category** | **Unit** | **Total potential impact** | **Normalized Result** |
| --- | --- | --- | --- |
| Acidification | molc H+ eq | 0.028605 | 0.000509893 |
| Climate change | kg CO_2_ eq | 8.795993 | 0.001165032 |
| Freshwater ecotoxicity | CTUe | 172.1602 | 0.046032182 |
| Freshwater eutrophication | kg P eq | 0.004089 | 0.000625191 |
| Human toxicity, cancer effects | CTUh | 9.15E-07 | 0.073812769 |
| Human toxicity, non-cancer effects | CTUh | 2.1E-06 | 0.013526048 |
| Ionizing radiation | kBq U235 eq | 0.593641 | 0.002463243 |
| Land use | kg C deficit | 15.51355 | 2.98338E-06 |
| Marine eutrophication | kg N eq | 0.007584 | 0.000249467 |
| Resource use, energy carriers | MJ | 113.9745 | 0.001324064 |
| Resource use, minerals and metals | kg Sb eq | 5.04E-05 | 0.000792292 |
| Ozone depletion | kg CFC-11 eq | 1.67E-07 | 1.37134E-05 |
| Particulate matter/ Respiratory inorganics | kg PM2.5 eq | 0.004065 | 0.000801789 |
| Photochemical ozone formation | kg NMVOC eq | 0.027696 | 0.000611397 |
| Terrestrial eutrophication | molc N eq | 0.066677 | 0.000406565 |
| Water resource depletion | m3 water eq | -0.10527 | -0.001527816 |

**Table 23.** Total potential impact and normalized results for a dental extraction following a digital consent process (scenario B).

The total potential burden a dental extraction has in a university clinic/medical care center. Total impacts were normalized using the average expenditure of a person per year using normalization factors from ILCD 2011 Midpoint+ for most data points and EF3.1 factors for those impact categories retrieved from impact assessment methods IPCC 2021, CML v4.8 2016 and EF v3.1. The value 1 would represent the total consumption per year^1^

**Life cycle impact assessment results for one dental extraction with dental travel (supplementary analysis)**

| **Impact Category** | **Unit** | **Plastic** | **Isopropanol** | **Acrylonitrile** | **Electricity** | **Ethanol** | **Waste** | **Gas** | **Soap** |
| --- | --- | --- | --- | --- | --- | --- | --- | --- | --- |
| Acidification | molc H+ eq | 6.71E-06 | 0.000767 | 0.000263 | 0.002384 | 8.68E-05 | 2.9E-05 | 0.000121 | 0.000860 |
| Climate change | kg CO_2_ eq | 0.001634 | 0.176419 | 0.043552 | 1.137786 | 0.00691 | 0.044719 | 0.080236 | 0.208997 |
| Freshwater ecotoxicity | CTUe | 0.016511 | 1.352779 | 0.291939 | 14.159527 | 0.269501 | 6.180475 | 0.286248 | 2.657114 |
| Freshwater eutrophication | kg P eq | 9.25E-07 | 1.94E-05 | 6.35E-06 | 0.001638 | 2.08E-06 | 3.29E-06 | 4.08E-06 | 0.001378 |
| Human toxicity, cancer effects | CTUh | 1.38E-10 | 7.16E-09 | 1.73E-09 | 1.34E-07 | 4.49E-10 | 4.02E-09 | 7.27E-09 | 9.96E-09 |
| Human toxicity, non-cancer effects | CTUh | 3.7E-10 | 2.33E-08 | 4.75E-09 | 3.96E-07 | 1.09E-08 | 6.99E-08 | 3.35E-09 | 5.96E-08 |
| Ionizing radiation | kg Sb-Eq | 0.000625 | 0.0073 | 0.000552 | 0.271567 | 0.000111 | 8.47E-05 | 0.000577 | 0.004169 |
| Land use | kBq U235 eq | 0.001737 | 0.049222 | 0.015498 | 0.42923 | 0.162287 | 0.006603 | 0.041686 | 7.048171 |
| Marine eutrophication | kg C deficit | 1.24E-06 | 9.63E-05 | 0.000155 | 0.000804 | 3.71E-05 | 1.6E-05 | 4.1E-05 | 0.001024 |
| Resource use, energy carriers | MJ | 0.025297 | 5.079802 | 0.920338 | 12.209598 | 0.043263 | 0.039186 | 5.714346 | 0.714215 |
| Resource use, minerals and metals | kg N eq | 3.77E-09 | 8.03E-07 | 1.65E-07 | 9.39E-07 | 6.21E-08 | 8.08E-09 | 4.92E-08 | 7.76E-07 |
| Ozone depletion | kg CFC-11 eq | 5.84E-11 | 1.74E-09 | 3.14E-10 | 7.96E-09 | 4.74E-10 | 1.35E-10 | 1.31E-08 | 4.12E-09 |
| Particulate matter/Respiratory inorganics | kg PM2.5 eq | 5.51E-07 | 7.09E-05 | 2.06E-05 | 0.000150 | 5.26E-06 | 2.66E-06 | 1.05E-05 | 0.000165 |
| Photochemical ozone formation | kg NMVOC eq | 4.87E-06 | 0.000742 | 0.000109 | 0.001543 | 3.14E-05 | 3.58E-05 | 0.000504 | 0.000519 |
| Terrestrial eutrophication | molc N eq | 1.15E-05 | 0.001025 | 0.000812 | 0.005787 | 0.000362 | 0.000132 | 0.000439 | 0.003187 |
| Water resource depletion | m3 water eq | -5.18E-05 | -0.002291 | -0.000277 | 0.000219 | 0.000192 | -3.15E-05 | 8.71E-06 | 0.002321 |

| **Steam** | **Steel** | **Water** | **Polyester** | **Cotton** | **Large lorry** | **Small lorry** | **Sea freight** | **Tissue paper** | **Wastewater** |
| --- | --- | --- | --- | --- | --- | --- | --- | --- | --- |
| 0.004511 | 0.000224 | 2.91E-05 | 3.48E-06 | 8.57E-06 | 3.63E-07 | 6.39E-05 | 0.000260 | 3.13E-05 | 0.000458 |
| 1.63981 | 0.050697 | 0.005343 | 0.000675 | 0.000697 | 0.000166 | 0.030134 | 0.008748 | 0.006292 | 0.097475 |
| 3.340229 | 2.072477 | 0.136999 | 0.008774 | 0.021834 | 0.001513 | 0.289908 | 0.030509 | 0.217466 | 1.217967 |
| 0.000185 | 2.27E-05 | 3.54E-06 | 1.82E-07 | 4.96E-07 | 1.19E-08 | 2.56E-06 | 2.96E-07 | 2.96E-05 | 8.50E-05 |
| 4.3E-08 | 4.27E-08 | 6.41E-09 | 6.64E-11 | 7.28E-11 | 1.21E-11 | 2.61E-09 | 5.16E-10 | 1.62E-09 | 7.74E-09 |
| 1.14E-07 | 2.51E-08 | 3.56E-09 | 1.55E-10 | 2.69E-10 | 3.84E-11 | 6.81E-09 | 4.26E-10 | 5.46E-08 | 1.48E-07 |
| 0.040230 | 0.004186 | 0.00202 | 4.03E-05 | 4.43E-05 | 3.2E-06 | 0.000843 | 5.14E-05 | 0.001005 | 0.01387 |
| 0.586450 | 0.144527 | 0.005813 | 0.000628 | 0.006444 | 0.000314 | 0.044483 | 0.006744 | 0.010677 | 2.228261 |
| 0.000752 | 6.48E-05 | 5.72E-06 | 7.72E-07 | 1.18E-05 | 9.23E-08 | 1.5E-05 | 6.51E-05 | 0.000344 | 0.000155 |
| 22.102623 | 0.481886 | 0.061347 | 0.011740 | 0.006273 | 0.002327 | 0.412321 | 0.107037 | 0.053708 | 1.125677 |
| 5.32E-07 | 2.87E-07 | 2.80E-08 | 5.20E-09 | 3.19E-09 | 5.44E-10 | 1.31E-07 | 8.45E-09 | 3.10E-08 | 3.22E-07 |
| 4.29E-08 | 9.21E-10 | 1.50E-10 | 1.27E-09 | 6.13E-12 | 2.97E-12 | 5.40E-10 | 1.08E-10 | 7.09E-11 | 3.55E-09 |
| 0.000498 | 4.92E-05 | 3.43E-06 | 4.71E-07 | 5.57E-07 | 6.12E-08 | 8.9E-06 | 9.53E-06 | 3.43E-06 | 4.94E-05 |
| 0.003771 | 0.000174 | 1.88E-05 | 3.18E-06 | 2.72E-06 | 5.52E-07 | 9.25E-05 | 0.000194 | 2.10E-05 | 0.000278 |
| 0.007747 | 0.000512 | 5.44E-05 | 8.69E-06 | 3.28E-05 | 9.32E-07 | 0.000150 | 0.00072 | 9.87E-05 | 0.001091 |
| -0.06590 | -7.44E-05 | 0.002783 | -2.94E-06 | 6.14E-05 | -1.33E-07 | -3.87E-05 | -1.54E-06 | -4.36E-05 | -0.002457 |

**Table 24.** Life cycle impact assessment results for one dental extraction with dental travel (supplementary analysis).

**Supplementary Analysis. Figurative representation of Life Cycle Impact Assessment results for one dental extraction without dental travel**


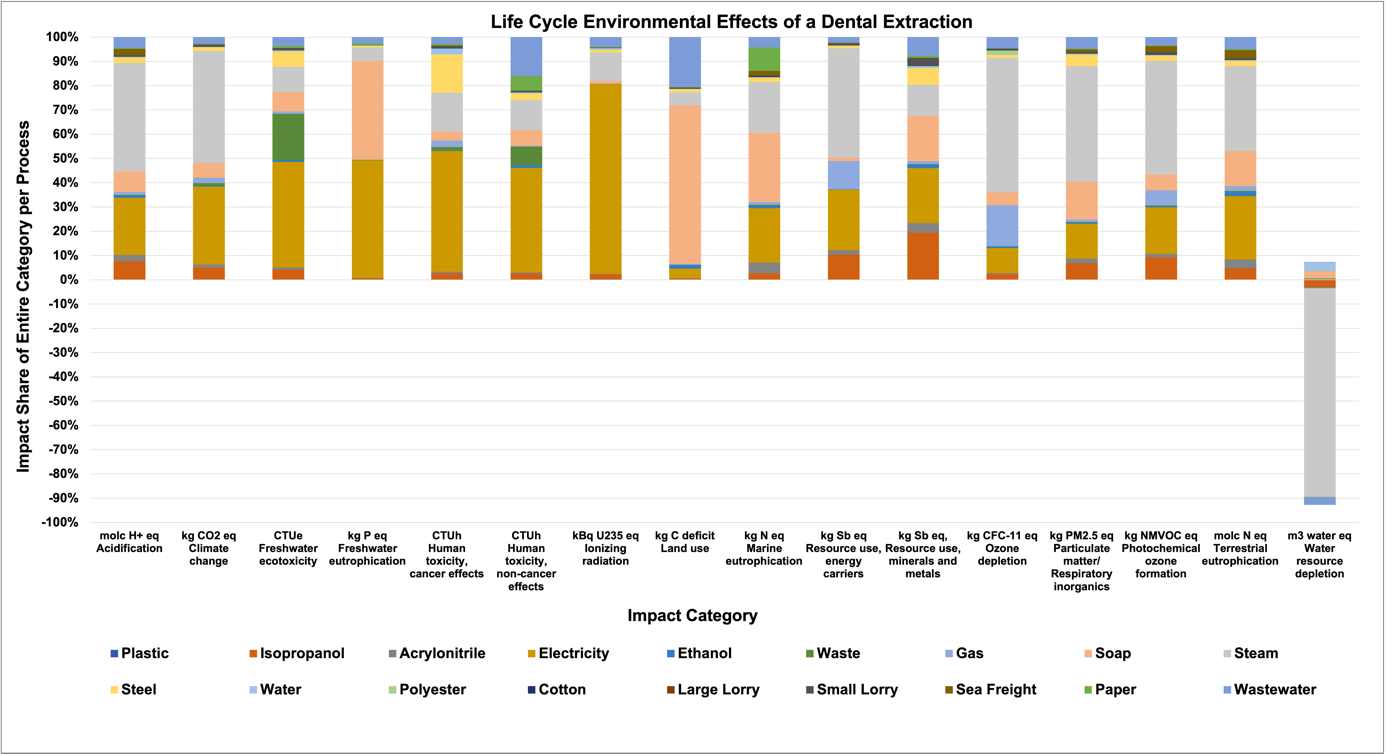


**Figure 7.** Supplementary Analysis. Life cycle impact assessment results for the dental extraction process without dental travel.

Total potential impact and normalized results for a dental extraction following a conventional consent process (supplementary analysis).

| **Impact Category** | **Unit** | **Total potential impact** | **Normalized Result** |
| --- | --- | --- | --- |
| Acidification | molc H+ eq | 0.010107 | 0.000180158 |
| Climate change | kg CO_2_-eq | 3.540291 | 0.000468913 |
| Freshwater ecotoxicity | CTUe | 32.55177 | 0.008703692 |
| Freshwater eutrophication | kg P eq | 0.003382 | 0.000517153 |
| Human toxicity, cancer effects | CTUh | 2.69E-07 | 0.021717117 |
| Human toxicity, non-cancer effects | CTUh | 9.21E-07 | 0.00594215 |
| Ionizing radiation HH | kBq U235 eq | 0.347278 | 0.001440989 |
| Land use | kg C deficit | 10.78878 | 2.07477E-06 |
| Marine eutrophication | kg N eq | 0.003589 | 0.000118051 |
| Resource use, energy carriers | MJ | 49.11098 | 0.000755554 |
| Resource use, minerals and metals | kg Sb eq | 4.16E-06 | 6.53314E-05 |
| Ozone depletion | kg CFC-11 eq | 7.73E-08 | 6.33985E-06 |
| Particulate matter/Respiratory inorganics | kg PM2.5 eq | 0.001049 | 0.000206892 |
| Photochemical ozone formation | kg NMVOC eq | 0.008046 | 0.000177611 |
| Terrestrial eutrophication | molc N eq | 0.02217 | 0.000135183 |
| Water resource depletion | m3 water eq | -0.06559 | -0.000951926 |

**Table 25.** Total potential impact and normalized results for a dental extraction following a conventional consent process (supplementary analysis).

The potential burden a dental extraction has in a university clinic/medical care center without dental travel. Total impacts were normalized using the average expenditure of a person per year using normalization factors from ILCD 2011 Midpoint+ for most data points and EF3.1 factors for those impact categories retrieved from impact assessment methods IPCC 2021, CML v4.8 2016 and EF v3.1. The value 1 would represent the total consumption per year.
